# Postnatal development of vasoactive intestinal polypeptide‐expressing GABAergic interneurons in mouse somatosensory cortex

**DOI:** 10.1111/apha.14265

**Published:** 2025-01-13

**Authors:** Clara A. Simacek, Sergei Kirischuk, Thomas Mittmann

**Affiliations:** ^1^ Institute for Physiology University Medical Centre of the Johannes Gutenberg University Mainz Mainz Germany

**Keywords:** active whisking, barrel field, development, *E*/*I* balance, interneuron, somatosensory cortex, VIP

## Abstract

**Aim:**

Despite dysfunctional vasoactive intestinal polypeptide‐positive interneurons (VIP‐INs) being linked to the emergence of neurodevelopmental disorders, the temporal profile of VIP‐IN functional maturation and cortical network integration remains unclear.

**Methods:**

Postnatal VIP‐IN development was traced with patch clamp experiments in the somatosensory cortex of *Vip‐IRES‐cre x tdTomato* mice. Age groups were chosen during barrel field formation, before and after activation of main sensory inputs, and in adult animals (postnatal days (P) P3–4, P8–10, P14–16, and P30–36).

**Results:**

Changes in passive and active membrane properties show a maturation towards accelerated signal integrations. Excitatory and inhibitory postsynaptic currents (EPSCs and IPSCs) showed progressive VIP‐IN integration into cortical networks, likely via synaptogenesis: mEPSC frequency increased before P8–10, while mIPSC frequency increased at P14–16. Only mIPSC kinetics became accelerated, and the *E*/*I* ratio of synaptic inputs, defined as a ratio of mEPSC to mIPSC charge transfer, remained constant throughout the investigated developmental stages. Evoked (e)EPSCs and (e)IPSCs showed increased amplitudes, while only eIPSCs demonstrated faster kinetics. eEPSCs and eIPSCs revealed a paired‐pulse facilitation by P14–16, indicating probably a decrease in the presynaptic release probability (*p*_*r*_) and a paired‐pulse depression in adulthood. eIPSCs also showed the latter, suggesting a decrease in *p*_*r*_ for both signal transmission pathways at this time point.

**Conclusions:**

VIP‐INs mature towards faster signal integration and pursue different strategies to avoid overexcitation. Excitatory and inhibitory synaptic transmission become stronger and shorter via different pre‐ and postsynaptic alterations, likely promoting the execution of active whisking.

## 1 INTRODUCTION

For enabling the central nervous system to be plastic throughout an organism's lifespan, neurons of primary sensory cortices first establish topographical maps of sensory modalities, which then undergo diverse structural and functional changes to establish and refine cortical networks.^1^, ^2^, ^3^, ^4^, ^5^, ^6^ A key driver for these reorganizing processes in the cortex is the onset of incoming sensory inputs, which mark the starting point of critical periods in early postnatal development and commence during, for example, eye and ear canal opening or the onset of active whisking behavior.^7^, ^8^, ^9^, ^10^, ^11^, ^12^ As excitatory and inhibitory connections undergo developmental reorganizations in parallel, keeping the overall cortical excitation and inhibition (*E*/*I*) in a physiological ratio appears to be a vital task during early cortex development.^13^, ^14^ By releasing the inhibitory neurotransmitter γ‐aminobutyric acid (GABA), GABAergic interneurons (INs) mediate inhibitory transmission, and among other functions, they are highly involved in postnatal cortical network refinement^15^ [for review, see Warm et al.^16^]. Malfunction of the GABAergic system can lead to an *E*/*I* imbalance from subcellular to cortex‐wide levels and, in turn, the emergence of neurological or neurodevelopmental disorders (NDDs) such as epilepsy, autism spectrum disorder (ASD), or schizophrenia^17^, ^18^, ^19^, ^20^, ^21^, ^22^ [for review, see Kirischuk^23^ and Tatti et al.^24^]. Despite GABAergic INs only comprising about 15%–20% of all cerebral neurons [for review, see Staiger et al.^25^], they are highly heterogeneous, and their classification takes features such as the expression of specific molecular markers, the location in the cortex, their morphology and their spiking pattern phenotypes into account^26^, ^27^, ^28^, ^29^, ^30^ [for review, see Staiger et al.^25^ and Feldmeyer et al.^31^]. Most frequently, GABAergic INs are subdivided into three main subpopulations based on the expression of certain molecular markers. Namely, parvalbumin (PV, ~40%), somatostatin (SST, ~30%). or the ionotropic serotonin receptor 5HT3aR (~30%). The latter can be further divided into a subpopulation co‐expressing the vasoactive intestinal polypeptide (VIP) [for review, see Tremblay et al.^32^ and Rudy et al.^33^]. Although VIP‐positive GABAergic INs constitute only ~15% of all GABAergic INs [for review, see Rudy et al.^33^], they are highly heterogeneous, as that is, demonstrated by varying morphologies and spiking pattern phenotypes.^34^, ^35^, ^36^, ^37^ The majority of cortical VIP‐positive INs (approx. 66%) are involved in a disinhibitory circuit motif in which they mainly inhibit SST‐expressing Ins, and by this, release downstream pyramidal neurons from their previous inhibition.^38^, ^39^, ^40^, ^41^, ^42^ Additionally, VIP‐positive INs of the somatosensory cortex convey a pivotal role in the developmental onset and general execution of active whisking behavior.^43^, ^44^, ^45^, ^46^ Developmental malfunction of VIP‐positive INs leads to several neuronal and behavioral abnormalities in different mouse models [for review, see Goff and Goldberg^47^]. For instance, dysregulated VIP interneurons during early postnatal development impair sensory processing and learning in adult mice, linking VIP interneuron malfunction to the emergence of NDD‐like symptoms.^48^ Developmental dysfunction of VIP interneurons is associated with ASD‐like behavior in a Dravet syndrome mouse model and with alterations in intrinsic electrophysiological properties, cortical activity, and behavioral phenotypes in a Rett syndrome mouse model.^49^ Even though these studies suggest a potential involvement of VIP‐positive interneurons in the emergence of severe NDDs,^50^ only little is known about the temporal profile of VIP‐positive interneuron functional maturation and their integration into the developing neocortex.

Our study aims to investigate how VIP interneurons change in function over development and how they integrate into the excitatory and inhibitory network of the somatosensory cortex during the first six postnatal weeks. We selected four time windows, namely postnatal day (P) 3–4: during the topographical barrel formation in the primary somatosensory cortex,^51^, ^52^ 8–10: before activation of main sensory inputs, P14–16: during and right after the activation of main sensory inputs,^10^, ^15^, ^53^ and P30–P36: adult animals. We conducted electrophysiological patch‐clamp experiments to investigate passive and active properties as well as synaptic inputs to VIP‐positive cells in layer 2/3 of the somatosensory cortex (VIP‐INs) of a *VIP‐IRES‐cre x tdTomato* mouse model. We present evidence that VIP‐INs undergo several developmental tunings: passive and active membrane properties evolve towards faster signal integration. Maturation of excitatory and inhibitory postsynaptic inputs demonstrates that VIP‐INs become increasingly integrated into both networks, while the excitatory and inhibitory signal transmission is kept in balance throughout development. Interestingly, both transmission pathways follow different developmental trajectories in terms of their temporal profile and processes of pre‐ and postsynaptic tunings to fulfill physiological functions. These changes presumably enable active whisking in a temporally precise manner via balanced excitation and inhibition, whilst avoiding sensory‐driven hypo‐ or hyperexcitation.

## 2 RESULTS

### 2.1 Maturation of passive membrane properties towards an accelerated signal‐integrating nature

**FIGURE 1:**
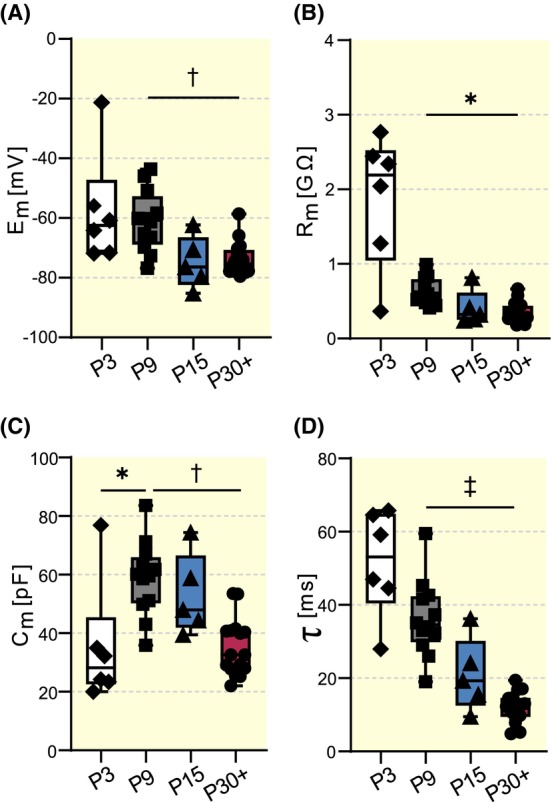
Developmental changes in passive membrane properties of VIP‐INs. (A–D) Statistical graphs showing developmental changes in and resting membrane potentials (*E*_*m*_, A), plasma membrane resistances (*R*_*m*_, B), plasma membrane capacitance (*C*_*m*_, C), and membrane time constant (*τ*, D) in VIP‐INs at P3, P9, P15, and P30+ in VIP‐INs. Number of cells: 6–16 (*p < 0.05, ^†^ *p* < 0.01, ^‡^ *p* < 0.001).

First, we examined putative developmental changes of passive membrane properties in VIP‐expressing neurons in L2/3 of the primary somatosensory cortex (VIP‐INs) across the four different postnatal stages (P3, P9, P15, and P30+). For this, we performed whole‐cell patch clamp recordings by using a K‐gluconate‐based intracellular solution (intracellular solution I, see Methods) and injecting a hyperpolarizing current step of −10 pA (Figure S1). The resting membrane potential (*E*_*m*_) became more hyperpolarized with increasing age (−58 ± 19, −61 ± 10, −75 ± 9, and −74 ± 5 mV at P3, P9, P15, and P30+, respectively). *E*_*m*_ was significantly different between P9 and P30+ (Kruskal‐Wallis, *p* = 0.0067, Figure 1A). The plasma membrane resistance *R*_*m*_ decreased significantly between P9 and 30+ from 626 ± 176 to 354 ± 136 MΩ (Kruskal‐Wallis, *p* = 0.0120, Figure 1B). The plasma membrane capacitance *C*_*m*_ showed a significant increase between P3 and P9 from 35 ± 21 to 59 ± 13 pF (Kruskal‐Wallis, *p* = 0.0100), while it significantly decreased between P9 and P30+ to 35 ± 10 pF (Kruskal‐Wallis, *p* = 0.0016, Figure 1C). The membrane time constant decreased significantly between P9 and P30+ from 36 ± 10 to 12 ± 4 ms (Kruskal‐Wallis, *p* = 0.0002, Figure 1D). For detailed statistical information, see Table S1.

The significant reductions in *E*_*m*_ and *R*_*m*_ suggest a decreased VIP‐IN excitability. The decline in the plasma membrane capacitance by P30+ is also reflected in a reduced membrane time constant, suggesting faster integration of incoming signals. Taken together with the reduction in excitability, this indicates a development of VIP‐INs towards fast signal‐integrating cells.

### 2.2 Developmental alterations in active membrane properties of VIP‐INs

**FIGURE 2:**
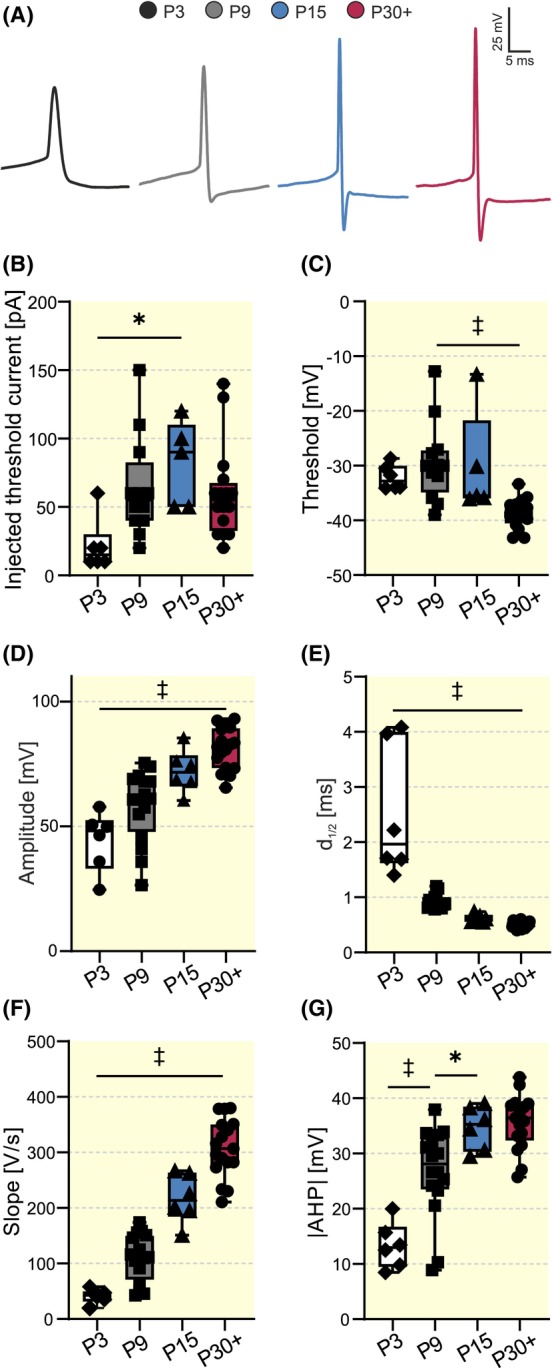
Developmental changes in active membrane properties of VIP‐INs. (A) Representative traces of the first action potentials (APs) at the injected threshold current across the four different age groups (P3, P9, P15, and P30+). (B–G) Statistical plots showing developmental changes of the injected threshold current (B), firing threshold (C), AP amplitude (D), AP half‐width (*d*_1/2_, E), AP rising slope (F), and amplitude of the after hyperpolarization portrayed in absolute values (G). Number of cells: 6–16 (*p < 0.05, ^‡^ *p* < 0.001).

Next, we examined developmental changes in the action potential (AP) generation of VIP‐INs by examining active membrane properties. For this, we used intracellular solution (I), applied a current step protocol, and considered the first elicited AP at the injected threshold current for further analysis (Figure 2A, Figure S1B).

APs demonstrated a gradual increase in their amplitude and the magnitude of the afterhyperpolarization (AHP) as well as a decrease in the half‐width and rising slope (Figure 2D,G), suggesting maturational processes towards AP discharges in higher frequencies. For detailed statistical information, see Table S1.

In parallel to the significant reduction in *R*_*m*_, the injected threshold current significantly increased between P3 and P15 from 22 ± 19 to 82 ± 31 pA (Kruskal‐Wallis, *p* = 0.0156, Figure 2B), showing a developmental decrease in membrane excitability. On the other hand, the more hyperpolarized *E*_*m*_ between P9 and P30+ (Figure 1) was accompanied by a significantly more hyperpolarized firing threshold between these time points (−29 ± 7 and −39 ± 3 mV at P9 and P30+, respectively, Kruskal‐Wallis, *p* = 0.0002, Figure 2C), thus favoring an increase in excitability.

A reduced membrane time constant leaves a smaller time window for signal integrations. However, this reduced excitability is partially compensated by a reduction in the AP threshold.

### 2.3 VIP‐IN integration into the excitatory and inhibitory networks of the barrel field

**FIGURE 3:**
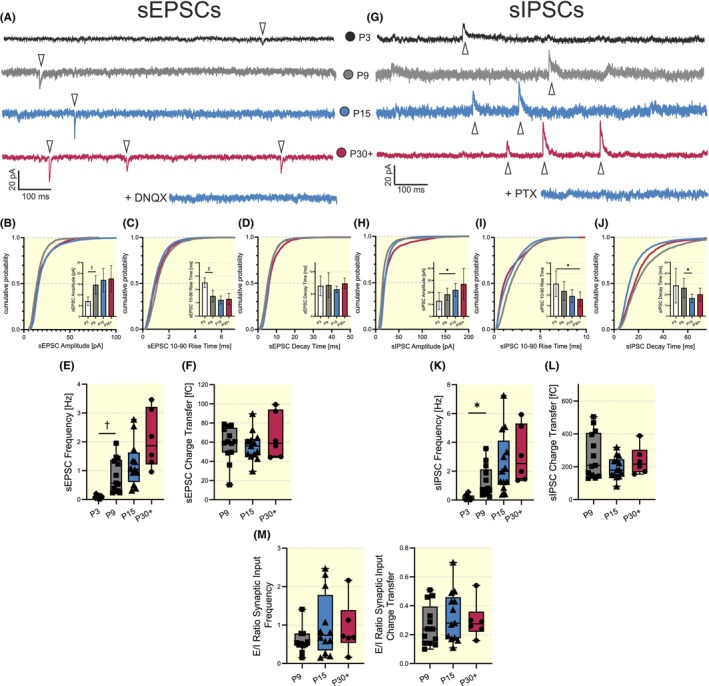
Developmental changes of spontaneous postsynaptic currents in VIP‐INs. (A) Representative traces of sEPSCs at P3, P9, P15, and P30+. Bath application of 20 μM DNQX completely abolished sEPSCs (bottom trace). (B–D) Cumulative probability distribution and bar plots demonstrate developmental changes of sEPSC amplitudes (B), sEPSC rise times (10%–90%, C), and sEPSC decay times (D). (E, F) Statistical plots of sEPSC frequency (E) and sEPSC charge transfer (F). (G) Representative traces of sIPSCs recorded from P3, P9, P15, and P30+ VIP‐INs. Bath application of 50 μM PTX abolished all sIPSCs. (H–J) Cumulative probability distribution and bar plots demonstrate developmental changes of sIPSC amplitudes (H), sIPSC rise times (10%–90%, I), and sIPSC decay times (J). (K, L) Statistical plots of sIPSC frequency (K) and sIPSC charge transfer (L). (M) Developmental changes in the *E*/*I* ratio of synaptic inputs are defined as the ratio of sEPSC frequency divided by sIPSC frequency (Left) and the *E*/*I* ratio of synaptic inputs is defined as the ratio of sEPSC charge transfer divided by sIPSC charge transfer (Right). Because of the low number of synaptic events (see frequency, E, K), P3 was excluded from the statistical analysis of the charge transfer (F, K) and the *E*/*I* ratios of synaptic events (M). Number of cells: 6–14. PTX, Picrotoxin (*p < 0.05, ^†^ *p* < 0.01, ^‡^ *p* < 0.001).

Neurons become integrated in cortical networks during development, receiving both excitatory and inhibitory synaptic inputs. Functional VIP‐IN excitability and signal‐integrating ability are dependent on both intrinsic properties and synaptic inputs. Therefore, we measured excitatory and inhibitory postsynaptic currents, EPSCs and IPSCs, respectively, in the barrel field of the somatosensory cortex. For this, we performed whole‐cell recordings on VIP‐INs at the four different developmental stages (P3, P9, P15, and P30+) and measured spontaneous EPSCs and IPSCs (sEPSCs and sIPSCs, respectively) (Figure 3A,G). Measurements were conducted with a Cs‐gluconate‐based intracellular solution (intracellular solution II; see Methods) to block K‐conductance. sEPSCs were recorded at −60 mV in the presence of D‐AP5 (25 μM) to block NMDA‐Rs. Bath application of DNQX (20 μM), an AMPA‐R antagonist, completely abolished all observed events, confirming their AMPA‐R origin (Figure 3A). sIPSCs were recorded at +10 mV. Bath application of PTX (50 μM) abolished all events, confirming that they were GABA_A_‐R‐mediated currents (Figure 3G).

Analysis of sEPSCs (Figure S1C) revealed significant developmental changes mainly between P3 and P9. For instance, the sEPSC amplitude significantly increased between P3 and P9 from 7 ± 2 to 15 ± 4 pA (Kruskal‐Wallis, *p* = 0.0052, Figure 3B). The 10–90 rise time demonstrated a significant shortening between P3 and P9 from 3 ± 0.4 to 2 ± 0.4 ms (ANOVA, *F* (3, 38) = 26.10, *p* < 0.0001, Figure 3C). The sEPSC decay time, however, remained unchanged across every age group (Kruskal‐Wallis, minimal *p*‐value 0.2987, Figure 3D). Because of no changes in sEPSC amplitude or kinetics in the time window between P9 to P30+, consequently no significant changes were detected in the sEPSC charge transfer (minimal *p*‐value 0.4807, ANOVA, *F* (2, 29) = 0.6875, Figure 3F). For detailed statistical information, see Table S2.

The mean amplitude of sIPSCs gradually increased between P3 and P15 from 13 ± 7 to 18 ± 5 pA (Kruskal‐Wallis, *p* = 0.0311, Figure 3H). Both the 10–90 rise time as well as the decay time revealed significant accelerations. For instance, the 10–90 rise time decreased between P3 and P30+ from 3 ± 1 to 2 ± 1 ms (Kruskal‐Wallis, *p* = 0.0177, Figure 3I) and the decay time decreased from 26 ± 9 to 17 ± 4 ms between P9 and P15 (Kruskal‐Wallis, *p* = 0.0279, Figure 3J). Despite the observed decreases in the rise time and decay time constants of sIPSCs, no developmental change in sIPSC charge transfer was detected (Kruskal‐Wallis, minimal *p*‐value 0.8307, Figure 3L), indicating that a potential increase in sIPSC amplitude can compensate for sIPSC shortening.

The frequencies of sEPSCs significantly increased between P3 and P9, with an increase from 0.1 ± 0.1 to 0.8 ± 0.6 Hz (Kruskal‐Wallis, *p* = 0.0069, Figure 3E). sIPSC frequency showed a significant increase within the same time window with corresponding values of 0.2 ± 0.2 and 1.3 ± 1.1 for P3 and P9, respectively (Kruskal‐Wallis, *p* = 0.0353, Figure 3K). This indicates a likely increase in the excitatory and inhibitory drive during postsynaptic maturation of neuronal networks. For detailed statistical information, see Table S2.

The *E*/*I* ratio of synaptic inputs was quantified in two ways: as the ratio of sEPSC frequency to sIPSC frequency and as the ratio of sEPSC charge transfer to sIPSC charge transfer. The P3 age group was excluded from this analysis, since the number of events (see frequency, Figure 3E,K) was so minimal that no meaningful interpretations could be drawn. Both the *E*/*I* ratio of synaptic input frequency and charge transfer failed to reveal any statistically significant change during development (Figure 3M, left: minimal *p*‐value 0.4316 (Kruskal‐Wallis); right: minimal *p*‐value 0.5133 (ANOVA, *F* (2, 29) = 0.6211)). This indicates a stable *E*/*I* ratio of the synaptic network across all investigated developmental stages.

### 2.4 Developmental trajectories of excitatory and inhibitory synaptic network integration significantly differ

Since spontaneous postsynaptic currents depend on both synaptic properties and the presynaptic AP‐dependent vesicle release machinery, we further investigated the synaptic inputs to VIP‐INs of the barrel field by recording miniature (m)EPSCs and (m)IPSCs in the presence of tetrodotoxin (1 μM), an antagonist of voltage‐gated Na^+^ channels (Figure 5A,G).

**FIGURE 4:**
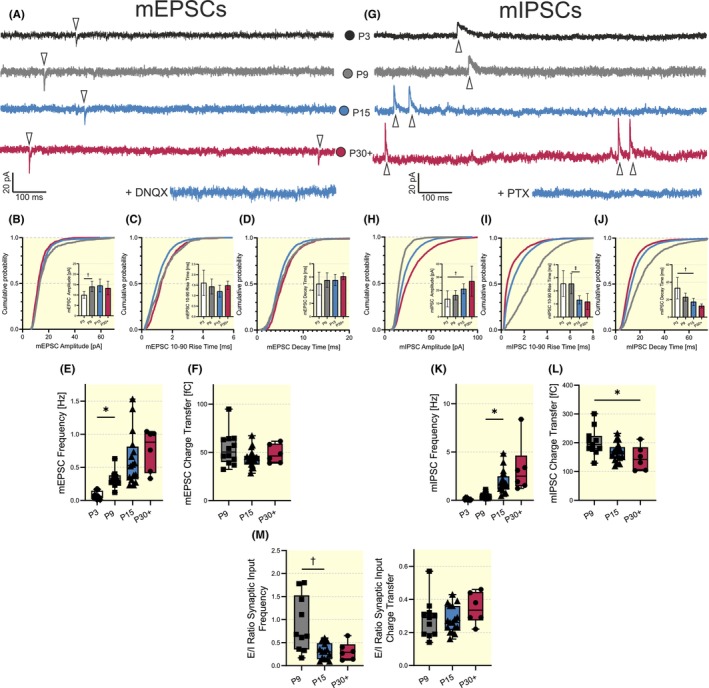
Developmental changes of miniature postsynaptic currents in VIP‐INs. (A) Representative traces of mEPSCs at P3, P9, P15, and P30+. Bath application of 20 μM DNQX completely abolished mEPSCs (the bottom trace). (B–D) Cumulative probability distribution and bar plots demonstrate developmental changes of mEPSC amplitudes (B), mEPSC rise times (10%–90%, C), and mEPSC decay times (D). (E, F) Statistical plots of mEPSC frequency (E) and mEPSC charge transfer (F). (G) Representative traces of mIPSCs recorded from P3, P9, P15 and P30+ VIP‐INs. Bath application of 50 μM PTX abolished all mIPSCs. (H–J) Cumulative probability distribution and bar plots demonstrate developmental changes of mIPSC amplitudes (H), mIPSC rise times (10%–90%, I), and mIPSC decay times (J). (K, L) Statistical plots of mIPSC frequency (K) and mIPSC charge transfer (L). (M) Developmental changes in the *E*/*I* ratio of synaptic inputs are defined as the ratio of mEPSC frequency divided by mIPSC frequency (Left) and the *E*/*I* ratio of synaptic inputs defined as the ratio of mEPSC charge transfer divided by mIPSC charge transfer (Right). Because of the low number of synaptic events (see frequency, E, K), P3 was excluded from the statistical analysis of the charge transfer (F, K) and the *E*/*I* ratios of synaptic events (M). Number of cells: 6–18 (*p < 0.05, ^†^ *p* < 0.01, ^‡^ *p* < 0.001).

Similar to sEPSCs (Figure 3), the mEPSC amplitude revealed a significant increase between P3 and P9 from 10 ± 2 to 14 ± 3 pA (ANOVA, *F* (3, 42) = 7.017, *p* = 0.0056, Figure 4B). Neither rise nor decay kinetics of mEPSCs demonstrated developmental changes (Kruskal‐Wallis, minimal *p*‐value 0.2072 for 10–90 rise time, minimal *p*‐value 0.0757 for decay time, Figure 4C,D). Consequently, for the time window between P9 and P30+, mEPSC charge transfer did not change significantly (minimal *p*‐value 0.1183, ANOVA, *F* (2, 33) = 2.088, Figure 4F). For detailed statistical information, see Table S3.

**FIGURE 5:**
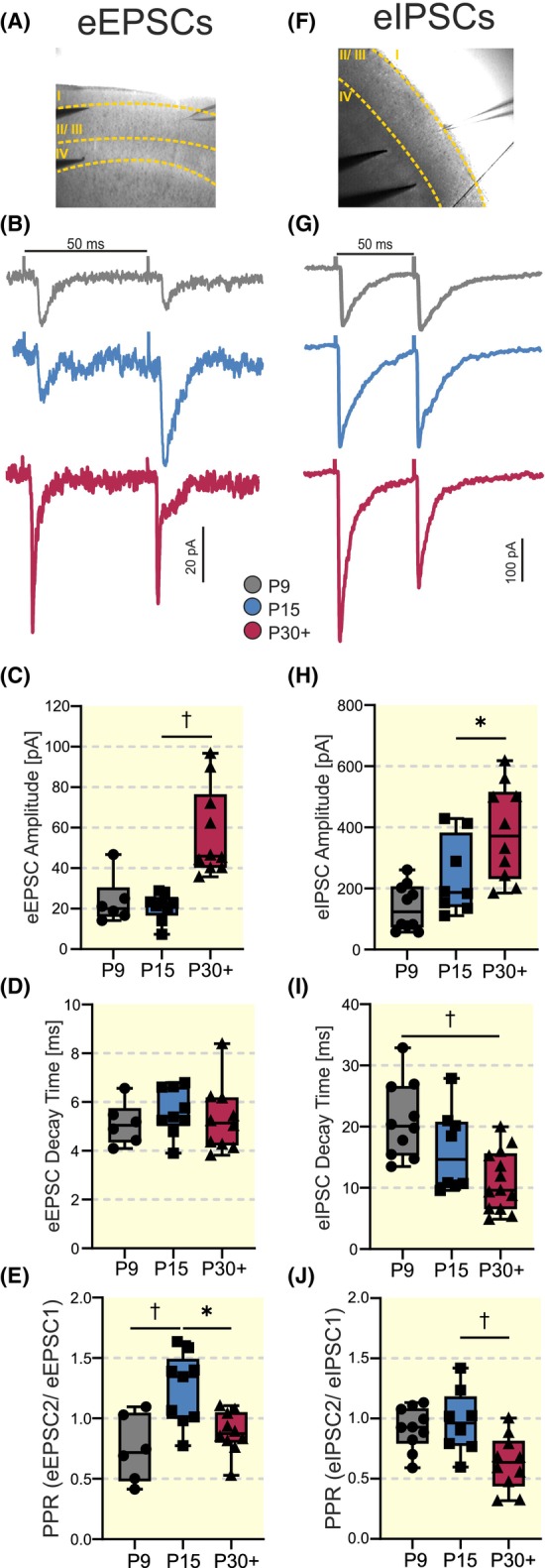
Developmental changes of functional properties of excitatory and inhibitory connections to VIP‐INs. (A, F) Example image showing stimulation and recording sites for eEPSCs and eIPSCs, respectively. (B, G) Representative traces of eEPSCs and eIPSCs elicited by a paired‐pulse stimulation at P9, P15, and P30+. (C–E, H–J) Statistical plots demonstrating developmental changes of the first eEPSC and eIPSC amplitude (C, H), first eEPSC and eIPSC decay time constant (D, I), and PPR (E, J) at P9, P15, and P30+. Number of cells: 6–10 (*p < 0.05, ^†^ *p* < 0.01).

mIPSCs showed several significant developmental alterations. Like sIPSCs, the mIPSC amplitude gradually increased from 14 ± 6 pA at P3 to 21 ± 4 pA at P15 (Kruskal‐Wallis, *p* = 0.0033, Figure 4H). Both rise time and decay time constants of mIPSCs demonstrated significant shortenings. Corresponding values for mIPSC rise times are 3 ± 1 for P9 and 1 ± 0.3 ms for P15, respectively (Kruskal‐Wallis, *p* = 0.0007, Figure 4I) and corresponding values for mIPSC decay time constants are 34 ± 12 for P3 and 18 ± 4 ms for P15 (Kruskal‐Wallis, *p* = 0.0014, Figure 4J). Despite increased mIPSC amplitudes, the acceleration of mIPSC kinetics resulted in a significant decrease of mIPSC charge transfer from 203 ± 47 fC at P9 to 147 ± 43 fC at P30+ (ANOVA, *F* (2, 31) = 4.751, *p* = 0.0211, Figure 5L). Both the frequency of mEPSCs and mIPSCs exhibited a developmental increase. mEPSC frequency increased from 0.08 ± 0.06 at P3 to 0.3 ± 0.1 Hz at P9 (Kruskal‐Wallis, *p* = 0.0323, Figure 4E) and mIPSC frequency increased significantly between P9 and P15 (0.5 ± 0.3 at P9 to 2 ± 1 Hz at P15, Kruskal‐Wallis, *p* = 0.0124, Figure 4K). For detailed statistical information, see Table S3.

The *E*/*I* ratio of synaptic input frequency revealed a shift towards the inhibitory side between P9 and P15 from 0.9 ± 0.6 to 0.3 ± 0.2 (Kruskal‐Wallis, *p* = 0.0090, Figure 4M left panel). However, the *E*/*I* ratio, defined as the ratio of mEPSC and mIPSC charge transfers, in which mIPSC kinetics are taken into account, remains in balance (minimal *p*‐value 0.2982, ANOVA, *F* (2, 31) = 1.223, Figure 5M, right panel). This indicates that despite a proportionally larger increase in the temporal precision of delivered inhibitory signals, the overall delivered charge of excitatory and inhibitory inputs remains constant throughout development.

In general, an increase in the frequency of mPSCs is indicative of presynaptic tunings, as it is suggested to predominantly reflect the number of synaptic contacts, while changes in mPSCs amplitude and kinetics reflect rather changes in the postsynaptic receptor number and/or subunit composition.^54^, ^55^, ^56^, ^57^

### 2.5 Excitatory and inhibitory connections mature both pre‐ and postsynaptically

Next, we examined developmental changes of presynaptic functional properties of glutamatergic and GABAergic connections onto VIP‐INs in the barrel field. For this, we performed whole‐cell patch clamp recordings. We used a K‐gluconate‐based solution to measure evoked EPSCs (eEPSCs) and a high Cl containing solution to measure evoked IPSCs (eIPSCs) (intracellular solutions I and III), respectively, (see Methods). To measure eEPSCs, a bipolar stimulation electrode was placed approximately. 500 μm medial to the barrel field while it was placed in L4 of the somatosensory cortex to electrically elicit evoked eIPSCs (Figure 5A,F). Both eEPSCs and eIPSCs were induced by maximal stimulation intensity, and a paired‐pulse protocol with an interstimulus interval of 50 ms was applied. As test experiments revealed a very low probability of eliciting either eEPSCs or eIPSCs (1 out of 24 cells, using 2 animals), the P3 group was excluded from these experiments. For detailed statistical information, see Table S4.

The amplitude of the first eEPSCs increased significantly between P15 and P30+ from 21 ± 7 to 57 ± 22 pA (Kruskal‐Wallis, *p* = 0.0017, Figure 5B,C). In agreement with sEPSCs and mEPSCs, eEPSC decay times remained constant throughout development (minimal *p*‐value 0.6903, ANOVA, *F* (2, 22) = 0.3640, Figure 5D).

The mean amplitude of ePSCs depends on several factors, including the presynaptic release probability. To probe this, we calculated the paired‐pulse ratio (PPR), that is the ratio of the mean amplitude of the second ePSCs to the mean amplitude of the first ePSCs (Figure S1D): the higher the initial release probability, the smaller the paired‐pulse ratio.^58^ Interestingly, the PPR of eEPSCs increased significantly between P9 and P15, while there was a significant decrease between P15 and P30+. Corresponding values are 0.7 ± 0.3, 1.2 ± 0.3, and 0.9 ± 0.2 for P9 (*n* = 6), P15 (*n* = 9), and P30+ (*n* = 10), respectively (ANOVA, *F* (2, 22) = 8.563, P9 vs. P15, *p* = 0.0026, and P15 vs. P30+, *p* = 0.0120, Figure 5E). This suggests a decrease in the release probability by P15, which is then elevated until P30+.

The first eIPSC amplitude increased significantly between P15 and P30+ (Figure 5G,H). Corresponding values are 238 ± 125 and 384 ± 156 pA at P15 and P30+, respectively (ANOVA, *F* (2, 25) = 10.05, *p* = 0.0496, Figure 5H). Similar to mIPSCs, the kinetics of eIPSCs were significantly faster, as reflected by a statistically significant decrease in eIPSC decay time constants between P9 and P30+ with corresponding values of 21 ± 6 and 11 ± 5 ms for P9 and P30+, respectively (ANOVA, *F* (2, 28) = 8.049, *p* = 0.0012, Figure 5I). PPRs were not significantly different at P9 and P15 (0.9 ± 0.2 and 1.0 ± 0.3 at P9 and P15, respectively; *p* = 0.8581). However, at P30+ the PPR decreased significantly to 0.6 ± 0.2 (*n* = 13, P15 vs. P30+: *p* = 0.0025, ANOVA, *F* (2, 28) = 8.988, Figure 5J), indicating probably an increase in presynaptic release probability.

In summary, the observed excitatory and inhibitory network integration demonstrates several developmental changes occurring at different time points. An increase in the number of release sites, probably due to synaptogenesis, takes place between P15 and P30+ for both the excitatory and inhibitory synaptic transmission. Additionally, there is an acceleration of inhibitory postsynaptic responses, presumably due to changes in postsynaptic GABA_A_‐R subunit composition. The presynaptic release probability in the excitatory signal transmission is first decreased between P9 and P15 and increased again by reaching adulthood. In contrast, the release probability of the inhibitory signal transmission remains constant until P15 and is elevated by P30+.

## 3 DISCUSSION

**FIGURE 6:**
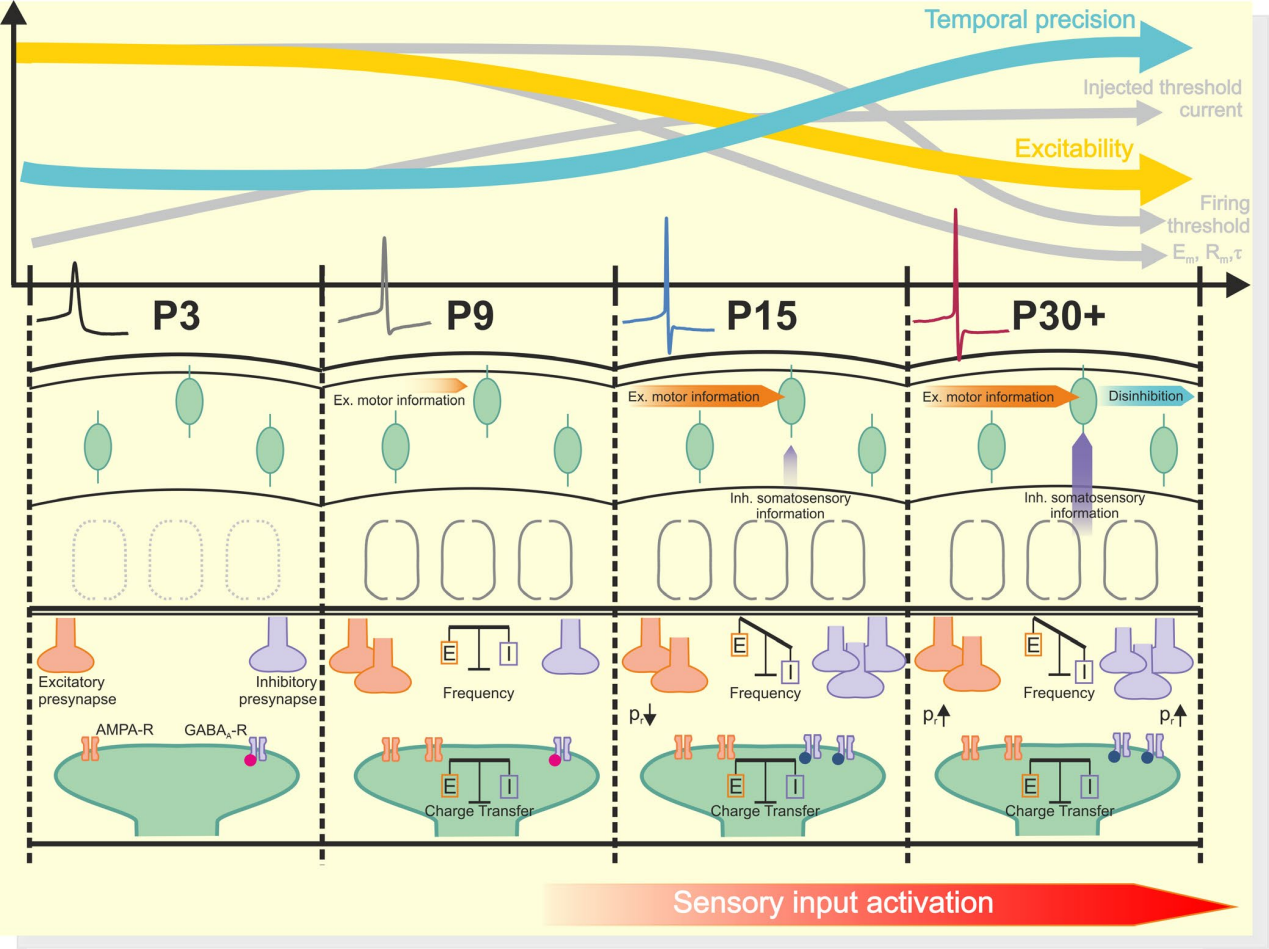
Summary figure of VIP‐IN development in mouse somatosensory cortex.

In this study we investigated the postnatal functional maturation and network integration of VIP‐INs in the developing somatosensory cortex. We have shown that VIP‐INs undergo several developmental changes during the first six postnatal weeks. Our results reveal (1) complex changes in passive and active membrane properties during the first six postnatal weeks, implying a functional maturation of VIP‐INs towards signal‐integration with high temporal precision; (2) with proceeding network refinement, VIP‐INs become increasingly integrated into both excitatory and inhibitory networks; (3) inhibitory synaptic currents become faster during development, enabling a temporally precise inhibition and giving support for stabilizing the *E*/*I* ratio of synaptic signal transmission; and (4) maturation of excitatory and inhibitory connections involves both pre‐ and postsynaptic mechanisms (Figure 6).

### 3.1 Functional maturation towards signal integration with high temporal precision

VIP‐INs demonstrate significant changes in both passive and active membrane properties during early postnatal development (see Table S1). Both resting membrane potential and membrane resistance decrease before P30+ and are accompanied with a continuous reduction of the membrane time constant during the investigated developmental period (Figure 1), indicating a decrease in excitability and a smaller time window for summation of synaptic inputs. Interestingly, the injected threshold current and the AHP amplitude (which might be interpreted as an intrinsic inhibition) increase already in the P15 group (Figure 2B,G). This suggests another, earlier step in decreasing the intrinsic excitability around the onset of active whisking, presumably to avoid sensory‐driven overexcitation. By P30+, however, the AP threshold becomes more negative, supporting faster integration of incoming sensory information (Figure 2C). Interestingly, the membrane capacitance increases between P3 and P9, while it decreases again until P30+ (Figure 1C). Since the membrane capacitance is calculated as the quotient between the membrane time constant and the membrane resistance, the increase of the membrane capacitance between P3 and P9 can be explained by the strong decrease of the membrane resistance within this time window (decrease by factor 3), while the membrane time constant remains on similar levels (Table S1).

In line with our data, very similar values have been reported for developmental changes of passive and active membrane properties between P8 and P15 for interneurons derived from the caudal ganglionic eminence (CGE), to which VIP‐positive interneurons belong,^59^ thus giving support to our findings. However, in contrast to our results, this study found a more depolarized resting membrane potential at P2/3 and could not elicit APs by the injection of depolarizing current steps. A possible reason for this might be that CGE‐derived interneurons not only comprise VIP‐positive cells but also the population of reelin‐ and calretinin‐positive interneurons.^60^ Moreover, maturing SST‐ and PV‐ INs are reported to show reductions in the membrane time constant, *R*_*m*_ and *E*_*m*_.^61^, ^62^, ^63^ This probably shows a common developmental trajectory in the maturation of passive membrane properties between the main classes of GABAergic INs towards a faster integrative function.

Our data show a developmental increase in AP amplitude and AHP amplitude as well as a shorter halfwidth and a faster rising slope (Figure 2D–G), which are functionally beneficial for promoting AP firing in higher frequencies. In contrast to the other two major classes of GABAergic INs,^61^, ^62^, ^63^ we failed to find a developmental increase in the firing frequency. However, our findings are in line with developing CGE‐derived interneurons^59^ and another, unpublished study investigating the development of VIP‐INs across similar age groups (Vagnioni et al., 2020, BioRxiv). However, VIP‐INs are highly heterogeneous in their firing pattern phenotypes,^36^, ^37^ and this inherent variability might affect and even hide statistical differences.

### 3.2 Integration of VIP‐INs into the developing neocortical circuitry

Synaptic inputs to VIP‐INs demonstrate significant developmental changes during the first six postnatal weeks (Tables S2 and S3). Both mEPSC and mIPSC frequencies show increases during the first three postnatal weeks (Figure 4), which is in line with results on PV‐INs in the developing somatosensory cortex.^64^ In general, increases in mEPSC and mIPSC frequencies suggest a proceeding innervation, probably via ongoing synaptogenesis, from excitatory and inhibitory neurons.^57^, ^65^ Since both mEPSC and mIPSC frequencies rise by P9 and P15, respectively, this indicates that both excitatory and inhibitory synaptogenesis have mostly been finished before sensory activation.

In general, the functional integration of VIP‐INs into excitatory networks demonstrates a complex temporal profile. Excitatory long‐range inputs from the motor cortex exist from as early as P5 on, and the number of connected cells increases until the onset of active whisking (Vagnioni et al., 2020, BioRxiv, unpublished). However, these projections appear to be rather functionally immature at early time points, as we failed to record any eEPSCs. In parallel, VIP‐INs receive excitatory drive from transient thalamocortical connections, which decline strongly after the onset of active whisking.^11^ The existence of temporal excitatory projections might explain why the frequency of both mEPSCs and sEPSCs remains stable from P9 on. One of the main inhibitory inputs to L2/3 VIP‐INs in the adult mouse somatosensory cortex is local, mainly in L4‐residing GABAergic INs.^11^, ^66^ It can be assumed that the increase in sIPSC and mIPSC frequency is due to an increased innervation from these local GABAergic‐INs. Interestingly, the observed increase of mIPSC frequency is accompanied by shortening of mIPSC duration and an increase in their amplitude, implying that this change is functionally a result of maturation of feed‐forward inhibition mediated by PV‐INs, residing in L4 of the somatosensory cortex.^67^

### 3.3 Maturation in inhibitory signal transmission balances *E/I* ratio of synaptic transmission

Increases in the aforementioned excitatory and inhibitory innervation can influence the *E*/*I* ratio of synaptic inputs. VIP‐INs exhibit a pronounced shift of the *E*/*I* ratio, defined as the ratio of mEPSC to mIPSC synaptic input frequency, towards inhibition before P15 (Figure 4M, left panel). This is due to a much stronger increase of mIPSC frequency in comparison to mEPSC frequency. However, there are no significant changes in the *E*/*I* ratio as defined by the ratio of mEPSC to mIPSC synaptic inputs charge transfer (Figure 4M, right panel). Hence, the *E*/*I* ratio of synaptic inputs of maturing VIP‐INs appears to be stable across each developmental stage. In line with our results, cortical PV‐INs also show a shift in the *E*/*I* ratio of synaptic input frequencies towards inhibition during postnatal maturation. However, they appear to have a higher excitatory drive at early developmental stages (P7–P15) as compared to VIP‐INs in similar age groups.^64^ In addition, it was shown for developing prefrontal pyramidal neurons that the *E*/*I* ratios of sPSC frequency and charge transfer can significantly vary between different layers of the same cortical region.^68^ It is worth mentioning that *E*/*I* ratio of synaptic input frequency mostly reflects presynaptic properties of inputs, while the *E*/*I* ratio of synaptic input charge transfer is dependent on both pre‐ and postsynaptic parameters. Taken together, this suggests that *E*/*I* ratios of synaptic transmission in terms of frequency and charge transfer can vary in a cell type‐, area‐ and layer‐specific manner during development and are adjusted both pre‐ and postsynaptically.

Interestingly, for the glutamatergic system, both sEPSCs (AP‐mediated responses) and mEPSCs (AP‐independent currents) demonstrate developmental changes only as an increase in their frequency (Tables S2 and S3). As EPSCs demonstrate significant alteration in amplitudes only between P3 and P9 and no significant changes in their kinetics, this data allows the suggestion that after P9 an increase in the number of synaptic contacts and/or inputs is the main mechanism of glutamatergic system maturation. In contrast, GABAergic projections to VIP‐INs demonstrate complex developmental alterations both in the number of synaptic inputs and postsynaptic responses, as demonstrated by both sIPSC and mIPSC data (Tables S2 and S3). A significant decrease of mIPSC charge transfer before P30+ due to accelerated mIPSC kinetics appears to be a powerful mechanism of balancing the *E*/*I* ratio of the synaptic transmission. Despite changes in mEPSC and mIPSC frequencies, the *E*/*I* ratio, defined as the ratio of mEPSC to mIPSC synaptic input charge transfer, remains consistent over VIP‐IN development. Changes in mPSC kinetics are in general associated with changes in receptor subunit composition.^54^, ^69^, ^70^ For GABA_A_‐receptor‐mediated currents, previous studies mentioned a role of the α_1_ subunit in the acceleration of mIPSC kinetics.^54^, ^55^ However, the question of whether the observed acceleration of mIPSC kinetics results from an α_1_‐subunit switch requires further experiments.

### 3.4 Maturation of excitatory and inhibitory signal transmission due to pre‐ and postsynaptic processes

To learn more about the properties of synaptic projections, we recorded evoked responses. Similar to mEPSCs, eEPSCs show an increase in amplitude at P30+ without any change in their kinetics (Figure 5A–E). This effect may be mediated by an increased number of release sites and/or an augmented presynaptic release probability. We have examined the latter by applying a paired‐pulse protocol.^71^ The PPR remained at similar values at P9 and P30+, however, it increased by P15, that is, before the activation of sensory inputs. This indicates that the observed increase in eEPSC amplitude at P30+ most probably reflects an increased number of release sites, rather than an increased release probability, since the latter would also imply a higher eEPSC amplitude at P9. The paired‐pulse facilitation at P15 might reflect a transient reduction of the presynaptic release probability before and probably during activation of main sensory inputs to the barrel cortex, which can be interpreted as a protective mechanism to avoid overexcitation. After the onset of main sensory activation, the number of synaptic release sites increases, and the excitatory signal transmission is additionally strengthened by an increase in the presynaptic release probability.

Similar to eEPSCs, developmental increases in eIPSC amplitudes suggest most probably an increased number of postsynaptic receptors (Figure 5H). In line with our results, PV‐INs of the prefrontal cortex show a developmental increase in the amplitude of unitary IPSCs, indicating an increase in the synaptic strength for inhibitory connections towards PV‐INs in the developing cortex.^62^ This supports an increase in eIPSC amplitude. Interestingly, both mEPSC and mIPSC amplitudes are statistically larger only at P9 and P15, respectively, while eEPSC and eIPSC amplitudes demonstrate continuous increase from P9 until P30+, with a significant rise between P15 and P30+. The difference may be explained by smaller mPSC amplitudes as compared to ePSC amplitudes, that is, small changes in mPSC amplitudes may be hidden by noise and become detectable in larger ePSCs. Since increases in mPSC and ePSC amplitude imply an increase in the number of postsynaptic receptors, the significant developmental step of eEPSC and eIPSC amplitude between P15 and P30+ suggests that the AMPA‐ and GABA_A_‐receptor number significantly increases after the onset of main sensory inputs, indicating that this development process is independent of sensory activation.

The PPR of eIPSCs remains constant between P9 and P15 and demonstrates strong reduction at P30+. Thus, around the onset of active whisking, the strength of inhibition is probably elevated due to an increase in the number of synaptic contacts/projections, and later the GABAergic transmission is additionally strengthened via an increase of release probability (Figure 5). Given that the main function of VIP‐INs is the inhibition of SST‐INs, this elevation of the presynaptic release probability in both the excitatory and inhibitory signal transmission, in combination with IPSC shortenings, enables a strong excitation, and by this inhibition of their downstream targets, while strong, short, and stimulus‐locked inhibitory inputs may act as pacesetters interrupting incoming excitation and by this disinhibition of downstream pyramidal neurons.

## 4 MATERIALS AND METHODS

### 4.1 Animals and ethical statement

Homozygous *Vip‐IRES‐cre* (Vip^tm1(cre)Zjh^, JAX Stock # 031628, The Jackson Laboratory, Bar Habor, USA) mice were crossed with homozygous tdTomato (*Ai14, B6.Cg‐Gt(ROSA)26Sor tm14(CAG‐tdTomato)Hze /J*, JAX Stock # 007914, The Jackson Laboratory^72^) reporter mice to obtain the V*ip‐IRES‐cre x tdTomato* reporter mouse line. To conduct histological and electrophysiological experiments, V*ip‐IRES‐cre x tdTomato* mice at three different postnatal stages were used: P3–P4 (referred to as P3, *N* = 7), P8–P10 (referred to as P9, *N* = 18), P14–P16 (referred to as P15, *N* = 16), and P30–P36 (referred to as P30+, *N* = 17), were used irrespective of their sex. Animals had constant access to food and water (ad libitum) and were kept under a standard 12 h day/night cycle at constant room temperature (23 ± 2°C). All experiments were designed to minimize the number of used animals. All experiments were performed in agreement with German and European laws of animal welfare in science (2010/63/EU).

### 4.2 Slice preparation

After deep anesthesia with 4% isoflurane, mice were decapitated. The brains were removed and transferred into ice‐cold oxygenated (95% O_2_, 5% CO_2_) cutting artificial cerebrospinal fluid (cACSF) containing (in mM): NaCl, 87; choline chloride, 37.5; KCl, 2.5; MgCl_2_ × 6 H_2_O, 7; CaCl_2_ × H_2_O, 0.5; NaH_2_PO_4_ × H_2_O, 1.25; NaHCO_3_, 25; and d‐glucose, 25; pH: 7.4 (reagents obtained from Carl Roth, Karlsruhe, Germany). Coronal brain slices of 300 μm thickness were cut using a vibratome (VT1200 S, Leica, Wetzlar, Germany). Slices containing somatosensory areas were incubated for a further 20 min in cACSF at 37°C and then transferred into regular ACSF (containing in mM: NaCl, 125; KCl, 2.5; MgCl_2_ × 6 H_2_O, 1; CaCl_2_ × H_2_O, 2; NaH_2_PO_4_ × H_2_O, 1.25; NaHCO_3_, 25; and d‐glucose, 25; pH: 7.4, reagents obtained from Carl Roth, Karlsruhe, Germany). Slices were incubated in the ACSF at RT for at least 45 min before usage.

### 4.3 Whole‐cell patch clamp recordings

Brain slices were transferred into a submerged recording chamber, mounted on an upright microscope (Olympus BX51WI, Tokyo, Japan). They were constantly perfused with oxygenated ACSF at a temperature of 37°C. L2/3 of the primary somatosensory cortex was visually identified by means of a lower magnification objective (5×, Olympus, Tokyo, Japan). Then, VIP‐INs were identified using a higher magnification objective (40×, Olympus, Tokyo, Japan) and a fluorescent lamp (U‐RTF‐L, Olympus, Tokyo, Japan) with a tdTomato filter cube (U‐M49010, Olympus, Tokyo, Japan). Electrophysiological recordings were performed by an Axopatch‐200B amplifier and Clampex 11.2 software (Molecular Devices, San José, CA, USA). Borosilicate glass pipettes (GB 150F‐8P; Science Products, Frankfurt, Germany) were pulled using a DMZ Zeitz‐Puller (Planegg, Germany). Pipette resistance ranged between 3 and 10 MΩ when filled with intracellular solution (with increasing age, a lower pipette resistance was chosen). Three different types of intracellular solutions were used for patch clamp recordings (in mM): (I) K‐gluconate, 140; KCl, 8; MgCl_2_ × 6 H_2_O, 2; Na_2_ATP, 4; Na_2_GTP hydrate, 0.3; Na_2_Phosphocreatin, 10; HEPES Potassium salt, 10, applied to study passive and active membrane properties. eEPSCs were recorded using intracellular solution (I) and adding N‐ethyl lidocaine (QX‐314), 2.5 to intracellularly block voltage‐gated Na^+^ channels; (II) Cs‐gluconate, 125; CsCl, 5; EGTA, 10; MgCl_2_, 2; Na_2_ATP, 2; Na_2_GTP, 0.4; HEPES, 10, used to record m(s)PSCs; (III) KCl, 130; NaCl, 5; EGTA, 5; HEPES, 20; CaCl_2_*2H_2_O, 0.5; Mg‐ATP, 2; Na‐GTP, 0.3; QX‐314, 2.5, used to record eIPSCs. pH was adjusted to a value of 7.3 using KOH for (I) and (III) and using CsOH for (II). The serial resistance (*R*_*s*_) and membrane resistance (*R*_*m*_) were monitored before and after each experiment, and cells were rejected when *R*_*s*_ exceeded 35 MΩ. All data was filtered at 2 kHz and digitized at 50 kHz using a Digidata‐1400 system with Clampex 11.1 software (Molecular Devices). In the current study, only VIP‐positive neurons in layer 2/3, that is, in the vicinity of layer 1, of the somatosensory cortex (VIP‐INs) were selected for recordings.

#### 4.3.1 Passive and active membrane properties

For recording passive and active membrane properties, intracellular solution (I) was used. The resting membrane potential was measured after achieving the whole‐cell configuration. For measurements in current clamp mode, cells were held at −70 mV. By injection of a hyperpolarizing current step of −10 pA for 1000 ms (20 repetitions per cell) and applying a mono‐exponential fit, *R*_*m*_ and the membrane capacitance (*C*_*m*_) were obtained (Figure S1A). Active membrane properties were measured by applying a current step protocol from −100 to 500 pA in steps of 10 pA. Each step lasted 1000 ms. The first action potential (AP) at the injected threshold current was used for analysis. The following properties were determined: the firing threshold, AP amplitude, halfwidth, AP rising slope and amplitude of the afterhyperpolarisation (Figure S1B). For analyzing passive and active membrane properties, Clampfit 11 software (Molecular Devices, San José, CA, USA) was used.

#### 4.3.2 Spontaneous and miniature postsynaptic currents

For measuring spontaneous excitatory and inhibitory postsynaptic currents (sEPSC and sIPSC, respectively), Cs‐containing intracellular solution (II) was used to block potassium conductance. Recordings were performed under voltage clamp conditions. VIP‐INs were kept at a holding potential of‐70 mV. For recording sEPSCs, cells were clamped at −60 mV, the Nernst‐reversal potential of GABA_A_‐receptor‐mediated currents. 2‐amino‐5‐phosphonovalerinans acid (DAP‐5, 25 μM), the antagonist for NMDA receptors, was added to the ACSF to isolate AMPA receptor‐mediated currents. Recordings of sIPSCs were conducted at a holding potential of +10 mV, the Nernst‐reversal potential of AMPA‐receptor‐mediated currents, to measure GABA_A_‐receptor‐mediated currents. Serial resistance compensation was not applied. sEPSCs and sIPSCs were recorded for at least 2 min. Miniature excitatory and inhibitory postsynaptic currents (mEPSC and mIPSC, respectively) were recorded in the same manner in the presence of tetrodotoxin (TTX, 1 μM), a blocker of voltage‐gated Na^+^‐channels. For analyzing spontaneous and miniature postsynaptic currents, MiniAnalysis software (Synaptosoft, Fort Lee, NJ, USA) was used (Figure S1C), and files were blinded so that the experimenter was not aware of the age group during analysis.

#### 4.3.3 Electrical stimulation

For recording electrically evoked excitatory (eEPSCs), a bipolar tungsten electrode was placed at least 500 μm more medial to the barrel field. The K‐gluconate‐containing intracellular solution (I) was used, and QX‐314 (2.5 mM) was added to both solutions to intracellularly block voltage‐gated Na^+^‐channels. DAP‐5 (25 μM) was added to the ACSF to block NMDA‐receptor‐mediated currents. Patched VIP‐INs in L2/3 were held at −60 mV. To record electrically evoked inhibitory postsynaptic currents (eIPSCs), a bipolar tungsten electrode was placed in L4 of the somatosensory cortex, and the high Cl^−^ intracellular solution (III) was used. QX‐314 (2.5 mM) was added to both solutions to intracellularly block voltage‐gated Na^+^ channels, and 6,7‐dinitroquinoxaline‐2,3‐dione (DNQX, 20 μM) and DAP‐5 (25 μM) were added to the ACSF to block excitatory synaptic activity. eIPSCs were measured at a holding potential of −70 mV.

The maximum stimulation intensity was determined by applying short electric pulses of 20 ns by means of a stimulus isolator (A360, World Precision Instruments, USA) with intensities between 25 and 600 μA until the ePSC amplitude was saturated. Typical stimulus intensity was about 250–350 μA. The paired‐pulse protocol consisted of two pulses applied with an interstimulus interval of 50 ms. At least 5 pairs were applied for each cell. To obtain the amplitude of the second eIPSC, the decay of the first eIPSC was fitted with an exponential function. The amplitude of the second eIPSC was measured from the decay phase of the first eIPSC (Figure S1D). Paired‐pulse ratio (PPR) was calculated as the ratio of the mean amplitude of the second IPSC divided by the mean amplitude of the first IPSC. Data was analyzed using PeakCount software v3.2. The program uses a derivative‐crossing algorithm to detect postsynaptic currents and then presents them for visual inspection.

### 4.4 Immunohistochemistry

Mice of each age group (for P3, P9, P15, and P30+) were deeply anesthetized with 4% isoflurane (AbbVie, Wiesbaden, Germany). After decapitation, the whole brain was carefully removed and fixated overnight in 4% PFA (Carl Roth, Karlsruhe, Germany) at 4°C. Brains were then transferred to 30% sucrose in phosphate‐buffered saline (PBS) for 3 days and cut into 20 μm thick coronal slices by means of a cryostat (Leica CM1325; Leica Mikrosysteme, Wetzlar, Germany). The PFA‐fixed brain slices were then blocked and permeabilized for two hours at room temperature with a solution containing 7% normal donkey serum (017‐000‐121, Dianova, Hamburg, Germany) and 0.8% Triton in 0.01 M PBS. The sections were incubated for 3 days at 4°C in the primary antibodies (ABs) rabbit‐anti‐VIP (1:2000, #20077, ImmunoStar, Dietzenbach, Germany) and goat‐anti‐mCherry (1:100, AB0040, Sicgen, Cantanhede, Portugal) in a staining buffer containing 2% bovine serum albumin (001‐000‐161, Dianova) with 0.05% azide and 0.3% Triton in 0.01 M PBS. After washing in 0.01 M PBS, slices were incubated in the secondary ABs and 0.5 μg/mL DAPI (A4099.0005, AppliChem, Darmstadt) for two hours at RT in a staining solution containing 2% bovine serum albumin (001‐000‐161, Dianova) with 0.05% azide. Secondary ABs were AF488‐conjugated and raised against rabbit IgG (1:200, Alexa Fluor® 488‐AffiniPure *F*(ab′)2 Fragment Donkey Anti‐Rabbit IgG (H+L), 711‐546‐152, Dianova) and Cy3‐conjugated and raised against goat (1:200, 705‐165‐147, Dianova). After washing in 0.01 M PBS, slices were mounted in Fluoromount‐G (SouthernBiotech, Birmingham, USA) mounting medium.

Layer 2/3 (L2/3) of the somatosensory cortex was visually identified by means of the DAPI staining and neuroanatomical landmarks at 25× magnification using a confocal microscope (Zeis LSM 710, Carl Zeiss AG, Oberkochen, Germany). Z‐Stacks of 5 fields of view of the somatosensory cortex were recorded per age group and further analyzed with Fiji/ImageJ software. Maximum projections of the Z‐Stacks and regions of interest (ROIs) from the cell somata were generated in the tdTomato and VIP channel. Cell density of cell somata in the VIP channel, tdTomato channel and the colocalized channels was determined. Soma areas were drawn from the ROI area of colocalized somata.

Cre recombinase expression can vary in an age‐dependent manner. For this we conducted immunohistochemical stainings in all four age groups (P3, P9, P15, and P30+) with antibodies against VIP and tdTomato. The colocalization between the two signals revealed a homogeneous and sufficiently high colocalization, indicating that the expression of the cre recombinase was restricted to VIP‐positive cells (Figure S2A–D). Additionally, the cell density remained constant throughout the four age groups (Figure S2E).

### 4.5 Data evaluation and statistics

All data was further analyzed using Excel 2021 (Microsoft, USA), and GraphPad Prism software (San Diego, CA, USA) was used for statistical evaluation. Results are given as mean ± standard deviation (SD). For all parameters, a Shapiro–Wilk test was applied to test for normality, and a Bartlett's test was applied to test for homoscedasticity. Given both criteria were met, statistical analyses were conducted by applying one‐way ANOVAs with post‐hoc Tukey's tests for multiple comparisons. If one or both criteria were violated, Kruskal‐Wallis tests with post hoc Dunn's test for multiple comparisons were applied. Statistical significances are given in the text as the corrected *p*‐values, which are represented by the symbols *p < 0.05; ^†^ *p* < 0.01; ^‡^ *p* < 0.001.

## 5 CONCLUSIONS

With our study, we provide extensive insights into the functional maturation and network integration of developing VIP‐INs. Especially shortly before the onset of main sensory inputs, VIP‐INs undergo complex maturational processes. Both intrinsic membrane properties and the inhibitory synaptic transmission are tuned for faster and more precise signal integrations. With this, VIP‐INs can mediate disinhibition to downstream pyramidal neurons in a temporally precise manner, thus functionally involving VIP‐INs of the somatosensory cortex in active whisking behavior. It appears a pivotal task of especially developing VIP‐INs during the onset of main sensory inputs is to pursue several strategies to avoid hypo‐ and/or hyperexcitation of the cortical network. By avoiding the emergence of an *E*/*I* imbalance in the network activity during this critical period of cortical development, VIP‐INs might contribute in this way to protections against NDD‐like pathologies.

## AUTHOR CONTRIBUTIONS

**Clara A. Simacek:** Conceptualization; methodology; data curation; writing – original draft; investigation; writing – review and editing; visualization; formal analysis. **Sergei Kirischuk:** Writing – review and editing; conceptualization; methodology; supervision. **Thomas Mittmann:** Writing – review and editing; conceptualization; funding acquisition; project administration; supervision; methodology; resources.

## FUNDING INFORMATION

This work is supported by the German Research Foundation to T.M. (DFG, CRC 1080, C02).

## CONFLICT OF INTEREST STATEMENT

The authors declare that they have no conflict of interest with this work.

## Supporting information


**Figure S1.**.

**Figure S2.**.

**Table S1.**.

## Data Availability

The data that support the findings of this study are available from the corresponding author upon reasonable request.
