# Supplementary figures and images for: Postnatal development of vasoactive intestinal polypeptide‐expressing GABAergic interneurons in mouse somatosensory cortex
Source: Acta Physiol (Oxf). 2025 Jan 13;241(2):e14265. doi: 10.1111/apha.14265 (PMC11726421; doi:10.1111/apha.14265)

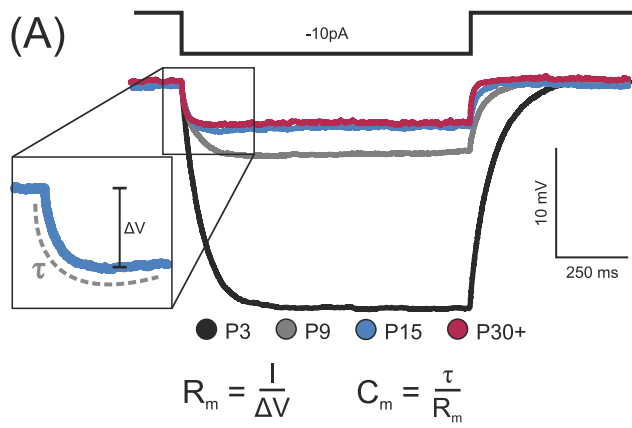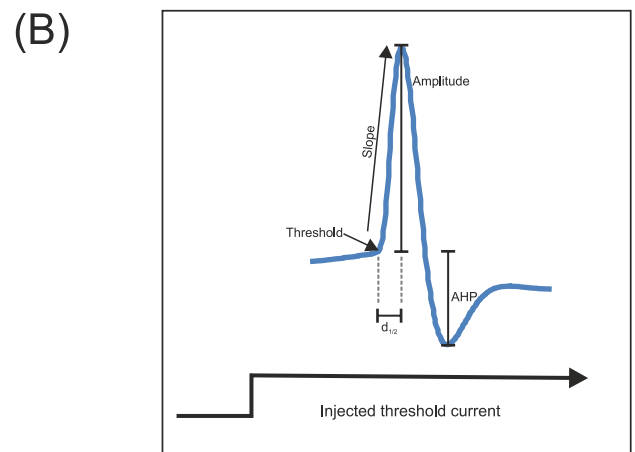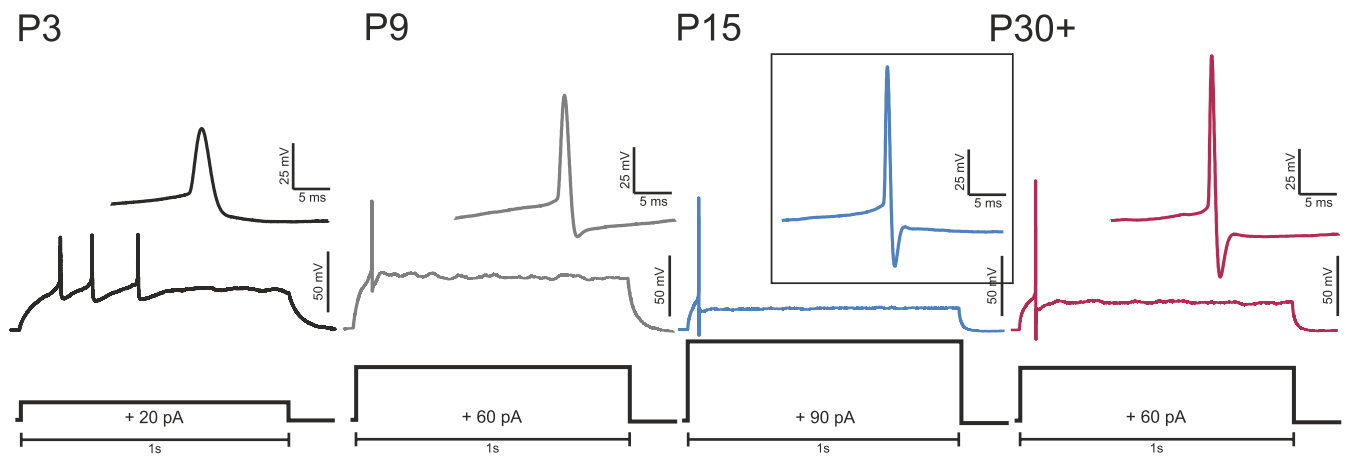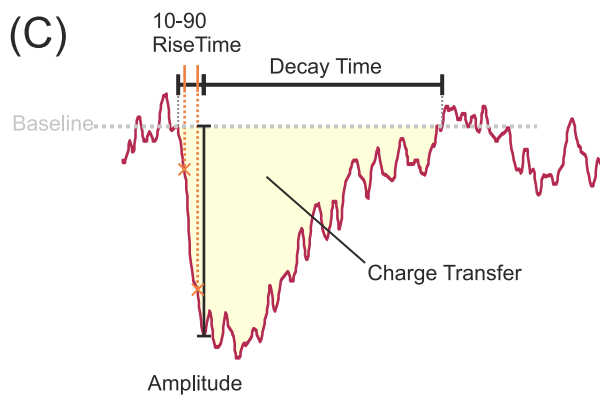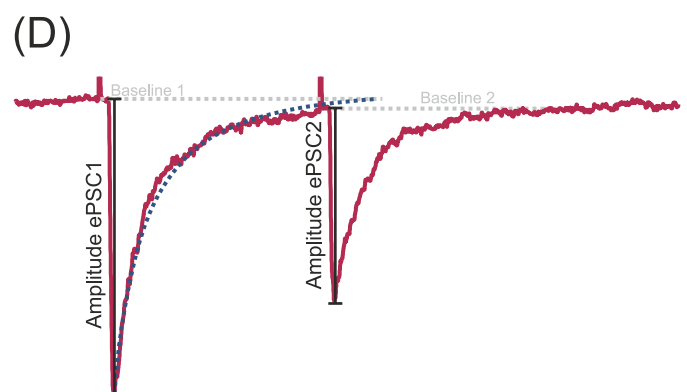

Supplement: Supplementary file 1 — Figure S1.. [file APHA-241-e14265-s001.pdf]

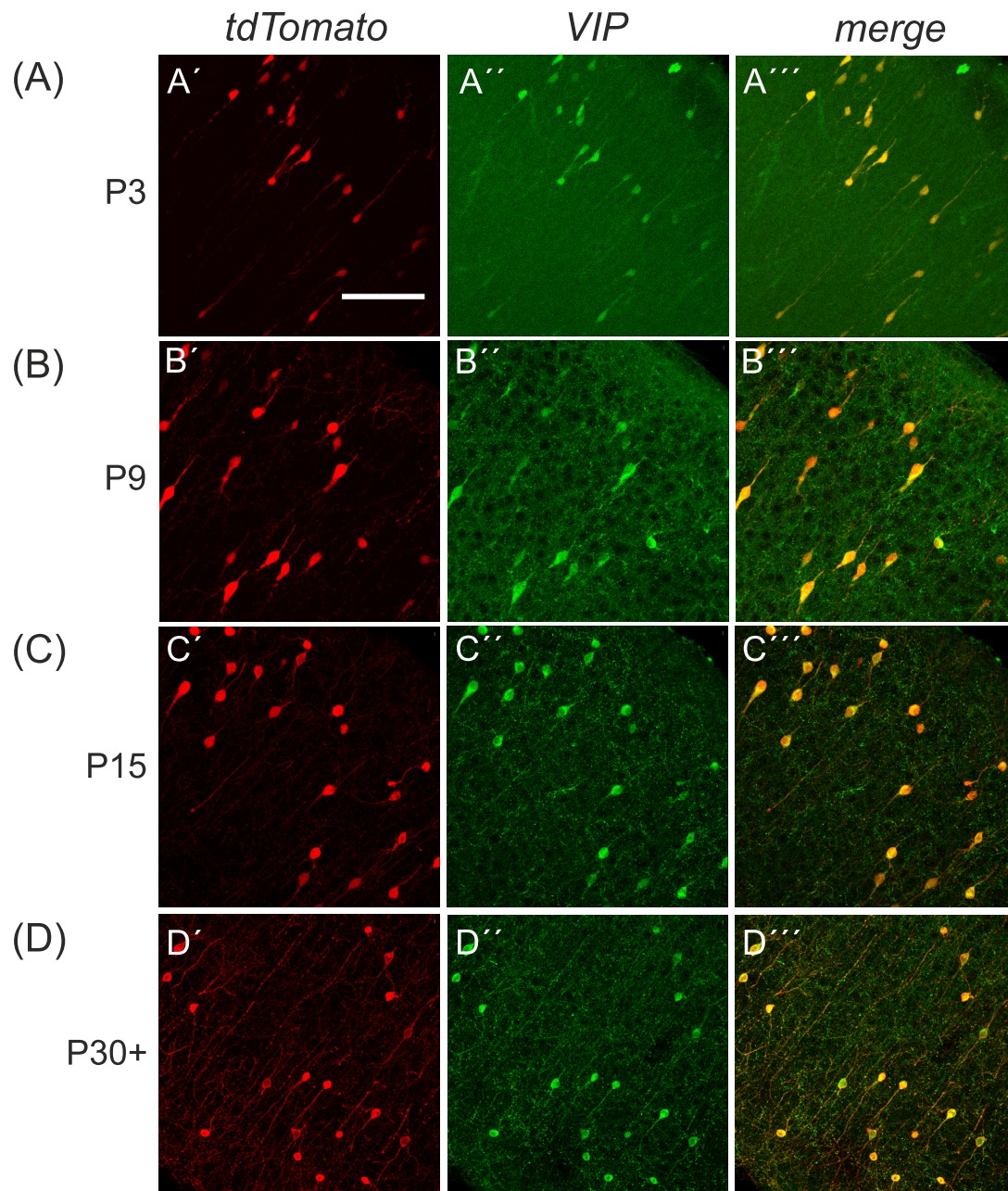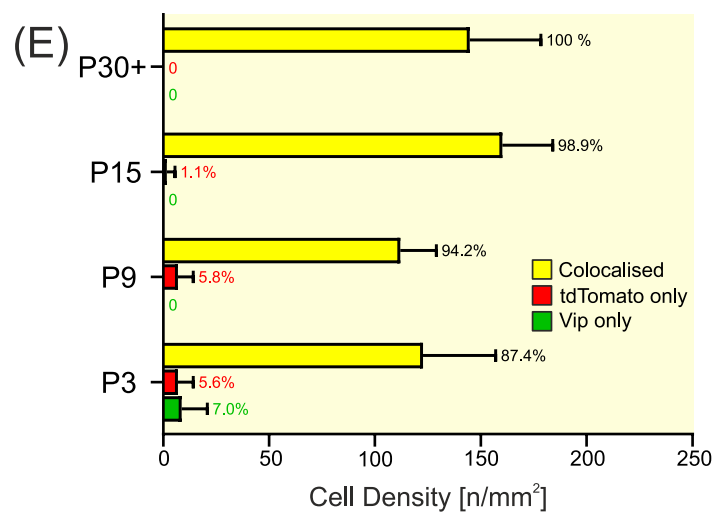

Supplement: Supplementary file 2 — Figure S2.. [file APHA-241-e14265-s003.pdf]
