# Supplementary material for: Postnatal development of vasoactive intestinal polypeptide‐expressing GABAergic interneurons in mouse somatosensory cortex
Source: Acta Physiol (Oxf). 2025 Jan 13;241(2):e14265. doi: 10.1111/apha.14265 (PMC11726421; doi:10.1111/apha.14265)
Supplement: Supplementary file 3 — Table S1.. [file APHA-241-e14265-s002.docx]

SUPPLEMENTARY TABLE 1: Developmental changes in passive and active membrane properties in VIP-INs.

|  | **P3** | **P9** | **P15** | **P30+** |  |
| --- | --- | --- | --- | --- | --- |
| *Passive membrane properties* | | | | | Statistical test |
| **Resting membrane potential E_m_ [mV]** | -57.6 ± 18.8  N = 6 | -60.9 ± 10.2  N = 12 | -74.8 ± 8.8  N = 5 | -73.6 ± 5.4  N = 16 | Kruskal-Wallis with post hoc Dunn’s test |
| **P3 vs. P9**  p = 0.9650  Adjusted  p > 0.9999 | **P3 vs. P15**  p = 0.0238  Adjusted  p = 0.1430 | **P3 vs. P30+**  p = 0.0082  **Adjusted**  **p = 0.0490*** | **P9 vs. P15**  p = 0.0114  Adjusted  p = 0.0685 | **P9 vs. P30+**  p = 0.0011  **Adjusted**  **p = 0.0067 †** | **P15 vs. P30+**  p = 0.8422  Adjusted  p > 0.9999 |
| **Membrane resistance R_m_ [GΩ]** | 1873 ± 895.8  N = 6 | 625.7 ± 176.4  N = 12 | 410.2 ± 236.5  N = 5 | 353.7 ± 136.0  N = 16 | Kruskal-Wallis with post hoc Dunn’s test |
| **P3 vs. P9**  p = 0.2061  Adjusted  p > 0.9999 | **P3 vs. P15**  p = 0.0053  **Adjusted**  **p = 0.0316 *** | **P3 vs. P30+**  p = 0.0002  **Adjusted**  **p = 0.0009 ‡** | **P9 vs. P15**  p = 0.0469  Adjusted  p = 0.2816 | **P9 vs. P30+**  p = 0.002  **Adjusted**  **p = 0.0120 *** | **P15 vs. P30+**  p = 0.8106  Adjusted  p > 0.9999 |
| **Membrane capacitance C_m_ [pF]** | 35.3 ± 21.2  N = 6 | 58.7 ± 12.7  N = 12 | 53.0 ± 13.9  N = 5 | 35.2 ± 9.6  N = 16 | Kruskal-Wallis with post hoc Dunn’s test |
| **P3 vs. P9**  p = 0.0017  **Adjusted**  **p = 0.0100 *** | **P3 vs. P15**  p = 0.0402  Adjusted  p = 0.2411 | **P3 vs. P30+**  p = 0.7084  Adjusted  p > 0.9999 | **P9 vs. P15**  p = 0.5366  Adjusted  p > 0.9999 | **P9 vs. P30+**  p = 0.0003  **Adjusted**  **p = 0.0016 †** | **P15 vs. P30+**  p = 0.0379  Adjusted  p = 0.2276 |
| **Membrane time constant τ [ms]** | 51.5 ± 14.5  N = 6 | 36.2 ± 10.4  N = 12 | 20.9 ± 10.1  N = 5 | 12.0 ± 4.1  N = 16 | Kruskal-Wallis with post hoc Dunn’s test |
| **P3 vs. P9**  p = 0.2794  Adjusted  p > 0.9999 | **P3 vs. P15**  p = 0.0190  Adjusted  p = 0.1137 | **P3 vs. P30+**  p < 0.0001  **Adjusted**  **p < 0.0001 ‡** | **P9 vs. P15**  p = 0.0983  Adjusted  p = 0.5897 | **P9 vs. P30+**  p < 0.0001  **Adjusted**  **p = 0.0002 ‡** | **P15 vs. P30+**  p = 0.1585  Adjusted  p = 0.9510 |
| *Active membrane properties* | | | | | Statistical test |
| **Injected threshold current [pA]** | 21.7 ± 19.4  N = 6 | 63.3 ± 36.8  N = 12 | 82.0 ± 31.1  N = 5 | 60.0 ± 33.5  N = 16 | Kruskal-Wallis with post hoc Dunn’s test |
| **P3 vs. P9**  p = 0.0099  Adjusted  p = 0.0592 | **P3 vs. P15**  p = 0.0026  **Adjusted**  **p = 0.0156 *** | **P3 vs. P30+**  p = 0.0091  Adjusted  p = 0.0549 | **P9 vs. P15**  p = 0.3160  Adjusted  p > 0.9999 | **P9 vs. P30+**  p = 0.9116  Adjusted  p > 0.9999 | **P15 vs. P30+**  p = 0.2608  Adjusted  p > 0.9999 |
| **Firing threshold [mV]** | -32.2 ± 2.2  N = 6 | -29.4 ± 7.2  N = 12 | -30.2 ± 9.8  N = 5 | -38.8 ± 2.6  N = 16 | Kruskal-Wallis with post hoc Dunn’s test |
| **P3 vs. P9**  p = 0.7700  Adjusted  p > 0.9999 | **P3 vs. P15**  p = 0.8280  Adjusted  p > 0.9999 | **P3 vs. P30+**  p = 0.0023  **Adjusted**  **p = 0.0139 *** | **P9 vs. P15**  p = 0.6018  Adjusted  p > 0.9999 | **P9 vs. P30+**  p < 0.0001  **Adjusted**  **p = 0.0002 ‡** | **P15 vs. P30+**  p = 0.0096  Adjusted  p = 0.0577 |
| **Amplitude [mV]** | 44.2 ± 12.0  N = 6 | 58.2 ± 14.2  N = 16 | 72.2 ± 8.6  N = 6 | 81.0 ± 8.3  N = 17 | One-way ANOVA with post hoc Tukey’s test  F (3, 41) = 20.66 |
| **P3 vs. P9**  p = 0.0133  Adjusted  p = 0.0613 | **P3 vs. P15**  p = 0.0001  **Adjusted**  **p = 0.0006 ‡** | **P3 vs. P30+**  p < 0.0001  **Adjusted**  **p < 0.0001 ‡** | **P9 vs. P15**  p = 0.0132  Adjusted  p = 0.0609 | **P9 vs. P30+**  p < 0.0001  **Adjusted**  **p < 0.0001 ‡** | **P15 vs. P30+**  p = 0.1046  Adjusted  p = 0.3577 |
| **Halfwidth d_1/2_ [ms]** | 2.5 ± 1.2  N = 6 | 0.9 ± 0.1  N = 15 | 0.6 ± 0.1  N = 6 | 0.5 ± 0.1  N = 17 | Kruskal-Wallis with post hoc Dunn’s test |
| **P3 vs. P9**  p = 0.0904  Adjusted  p = 0.5425 | **P3 vs. P15**  p = 0.0024  **Adjusted**  **p = 0.0144 *** | **P3 vs. P30+**  p < 0.0001  **Adjusted**  **p < 0.0001 ‡** | **P9 vs. P15**  p = 0.0530  Adjusted  p = 0.3179 | **P9 vs. P30+**  p < 0.0001  **Adjusted**  **p < 0.0001 ‡** | **P15 vs. P30+**  p = 0.1203  Adjusted  p = 0.7218 |
| **Slope [V/s]** | 40.9 ± 12.9 | 110.3 ± 42.1 | 217.2 ± 44.1 | 307.4 ± 52.6 | Kruskal-Wallis with post hoc Dunn’s test |
| **P3 vs. P9**  p = 0.1174  Adjusted  p = 0.7043 | **P3 vs. P15**  p = 0.0044  **Adjusted**  **p = 0.0265 *** | **P3 vs. P30+**  p < 0.0001  **Adjusted**  **p < 0.0001 ‡** | **P9 vs. P15**  p = 0.0618  Adjusted  p = 0.3711 | **P9 vs. P30+**  p < 0.0001  **Adjusted**  **p < 0.0001 ‡** | **P15 vs. P30+**  p = 0,0921  Adjusted  p = 0.5524 |
| **Afterhyperpolarisation AHP [mV]** | 13.3 ± 4.1  N = 6 | 26.7 ± 8.1  N = 16 | 34.7 ± 4.0  N = 6 | 35.7 ± 4.9  N = 17 | One-way ANOVA with post hoc Tukey’s test  F (3, 41) = 22.32 |
| **P3 vs. P9**  p < 0.0001  **Adjusted**  **p = 0.0002 ‡** | **P3 vs. P15**  p < 0.0001    **Adjusted**  **p < 0.0001 ‡** | **P3 vs. P30+**  p < 0.0001  **Adjusted**  **p < 0.0001 ‡** | **P9 vs. P15**  p = 0.0096  **Adjusted**  **p = 0.0456** * | **P9 vs. P30+**  p = 0.0001  **Adjusted**  **p = 0.0008 ‡** | **P15 vs. P30+**  p = 0.7298  Adjusted  p = 0.9853 |

SUPPLEMENTAYR TABLE 2: Developmental changes in sEPSCs ans sIPSCs of VIP-INs.

|  | **P3** | **P9** | **P15** | **P30+** |  |
| --- | --- | --- | --- | --- | --- |
| *sEPSCs* | | | | | Statistical test |
| **Amplitude [pA]** | 7.1 ± 2.0  N = 10 | 14.7 ± 4.3  N = 13 | 16.9 ± 5.4  N = 13 | 17.6 ± 6.1  N = 6 | Kruskal-Wallis with post hoc Dunn’s test |
| **P3 vs. P9**  p = 0.0009  **Adjusted**  **p = 0.0052 †** | **P3 vs. P15**  p < 0.0001  **Adjusted**  **p = 0.0003 ‡** | **P3 vs. P30+**  p = 0.0005  **Adjusted**  **p = 0.0028 †** | **P9 vs. P15**  p = 0.4241  Adjusted  p > 0.9999 | **P9 vs. P30+**  p = 0.4113  Adjusted  p > 0.9999 | **P15 vs. P30+**  p = 0.8522  Adjusted  p > 0.9999 |
| **10-90 Rise Time [ms]** | 2.5 ± 0.4  N = 10 | 1.5 ± 0.4  N = 13 | 1.2 ± 0.3  N = 13 | 1.3 ± 0.4  N = 6 | One-way ANOVA with post hoc Tukey’s test  F (3, 38) = 26.10 |
| **P3 vs. P9**  p < 0.0001  **Adjusted**  **p < 0.0001 ‡** | **P3 vs. P15**  p < 0.0001  **Adjusted**  **p < 0.0001 ‡** | **P3 vs. P30+**  p < 0.0001  **Adjusted**  **p < 0.0001 ‡** | **P9 vs. P15**  p = 0.0542  Adjusted  p = 0.2108 | **P9 vs. P30+**  p = 0.2431  Adjusted  p = 0.6395 | **P15 vs. P30+**  p = 0.6963  Adjusted  p = 0.9791 |
| **Decay Time [ms]** | 6.8 ± 2.2  N = 10 | 7.0 ± 2.8  N = 13 | 6.0 ± 0.8  N = 12 | 7.4 ± 1.2  N = 6 | Kruskal-Wallis with post hoc Dunn’s test |
| **P3 vs. P9**  p = 0.7846  Adjusted  p > 0.9999 | **P3 vs. P15**  p = 0.2954  Adjusted  p > 0.9999 | **P3 vs. P30+**  p = 0.3021  Adjusted  p > 0.9999 | **P9 vs. P15**  p = 0,.596  Adjusted  p = 0.9578 | **P9 vs. P30+**  p = 0.3971  Adjusted  p > 0.9999 | **P15 vs. P30+**  p = 0.0498  Adjusted  p = 0.2987 |
| **Frequency [Hz]** | 0.1 ± 0.06  N = 10 | 0.8 ± 0.6  N = 13 | 1.2 ± 0.8  N = 12 | 2.1 ± 1.0  N = 6 | Kruskal-Wallis with post hoc Dunn’s test |
| **P3 vs. P9**  p = 0.0011  **Adjusted**  **p = 0.0069 †** | **P3 vs. P15**  p < 0.0001  **Adjusted**  **p = 0.0003 ‡** | **P3 vs. P30+**  p < 0.0001  **Adjusted**  **p < 0.0001 ‡** | **P9 vs. P15**  p = 0.3445  Adjusted  p >0.9999 | **P9 vs. P30+**  p = 0.0390  Adjusted  p = 0.2343 | **P15 vs. P30+**  p = 0.2004  Adjusted  p > 0.9999 |
| **Charge Transfer [fC]** | 35.1 ± 15.0  N = 10 | 58.6 ± 18.1  N = 13 | 56.0 ± 15.1  N = 13 | 66.4 ± 23.8  N = 6 | One-way ANOVA with post hoc Tukey’s test  F (2, 29) = 0.6875 |
|  |  | **P9 vs. P15**  p = 0.7061  Adjusted  p = 0.9234 | **P9 vs. P30+**  p = 0.3934  Adjusted  p = 0.6654 | **P15 vs. P30+**  p = 0.2519  Adjusted  p = 0.4807 |  |
| *sIPSCs* | | | | | Statistical test |
| **Amplitude [pA]** | 12.9 ± 7.0  N = 9 | 18.3 ± 5.1  N = 12 | 22.0 ± 5.5  N = 14 | 27.0 ± 13.0  N = 6 | Kruskal-Wallis with post hoc Dunn’s test |
| **P3 vs. P9**  p = 0.1467  Adjusted  p = 0.8800 | **P3 vs. P15**  p = 0.0052  **Adjusted**  **p = 0.0311 *** | **P3 vs. P30+**  p = 0.0060  **Adjusted**  **p = 0.0363 *** | **P9 vs. P15**  p = 0.1587  Adjusted  p = 0.9519 | **P9 vs. P30+**  p = 0.1065  Adjusted  p = 0.6393 | **P15 vs. P30+**  p = 0.6049  Adjusted  p > 0.9999 |
| **10-90 Rise Time [ms]** | 3.0 ± 1.2  N = 9 | 2.4 ± 0.9  N = 13 | 1.9 ± 0.6  N = 14 | 1.6 ± 0.7  N = 6 | Kruskal-Wallis with post hoc Dunn’s test |
| **P3 vs. P9**  p = 0.1953  Adjusted  p > 0.9999 | **P3 vs. P15**  p = 0.0098  Adjusted  p = 0.0590 | **P3 vs. P30+**  p = 0.0029  **Adjusted**  **p = 0.0177 *** | **P9 vs. P15**  p = 0.1598  Adjusted  p = 0.9586 | **P9 vs. P30+**  p = 0.0417  Adjusted  p = 0.2499 | **P15 vs. P30+**  p = 0.3418  Adjusted  p > 0.9999 |
| **Decay Time [ms]** | 28.6 ± 15.9  N = 9 | 26.3 ± 8.7  N = 13 | 16.7 ± 3.8  N = 14 | 20.3 ± 5.9  N = 6 | Kruskal-Wallis with post hoc Dunn’s test |
| **P3 vs. P9**  p = 0.8008  Adjusted  p > 0.9999 | **P3 vs. P15**  p = 0.0217  Adjusted  p = 0.1302 | **P3 vs. P30+**  p = 0.3902  Adjusted  p > 0.9999 | **P9 vs. P15**  p = 0.0047  **Adjusted**  **p = 0.0279 *** | **P9 vs. P30+**  p = 0.2546  Adjusted  p > 0.9999 | **P15 vs. P30+**  p = 0.2793  Adjusted  p > 0.9999 |
| **Frequency [Hz]** | 0.2 ± 0.2  N = 9 | 1.3 ± 1.1  N = 13 | 2.6 ± 2.1  N = 13 | 3.2 ± 1.9  N = 6 | Kruskal-Wallis with post hoc Dunn’s test |
| **P3 vs. P9**  p = 0.0059  **Adjusted**  **p = 0.0353 *** | **P3 vs. P15**  p < 0.0001  **Adjusted**  **p = 0.0003 ‡** | **P3 vs. P30+**  p < 0.0001  **Adjusted**  **p = 0.0002 ‡** | **P9 vs. P15**  p = 0.1520  Adjusted  p = 0.9118 | **P9 vs. P30+**  p = 0.0496  Adjusted  p = 0.2974 | **P15 vs. P30+**  p = 0.4093  Adjusted  p > 0.9999 |
| **Charge Transfer [fC]** | 244.4 ± 215.8  N = 9 | 269.6 ± 136.8  N = 13 | 195.0 ± 65.9  N = 14 | 236.7 ± 85.7  N = 6 | Kruskal-Wallis with post hoc Dunn’s test |
|  |  | **P9 vs. P15**  p = 0.2769  Adjusted  p = 0.8307 | **P9 vs. P30+**  p = 0.9764  Adjusted  p > 0.9999 | **P15 vs. P30+**  p = 0.3745  Adjusted  p > 0.9999 |  |
| *E/I Ratio of synaptic inputs* | | | | | Statistical test |
| **E/I Ratio Synaptic Input Frequency** | 1.0 ± 0.7  N = 9 | 0.6 ± 0.3  N = 11 | 1.0 ± 0.8  N = 12 | 0.9 ± 0.7  N = 6 | Kruskal-Wallis with post hoc Dunn’s test |
|  |  | **P9 vs. P15**  p = 0.1438  Adjusted  p = 0.4316 | **P9 vs. P30+**  p = 0.1908  Adjusted  p = 0.5725 | **P15 vs. P30+**  p = 0.9142  Adjusted  p > 0.9999 |  |
| **E/I Ratio Synaptic Input Charge Transfer** | 0.3 ± 0.3  N = 9 | 0.3 ± 0.1  N = 13 | 0.3 ± 0.2  N = 13 | 0.3 ± 0.1  N = 6 | One-way ANOVA with post hoc Tukey’s test  F (2, 29) = 0.6211 |
|  |  | **P9 vs. P15**  p = 0.2744  Adjusted  p = 0.5133 | **P9 vs. P30+**  p = 0.6382  Adjusted  p = 0.8835 | **P15 vs. P30+**  p = 0.6847  Adjusted  p = 0.9117 |  |

SUPPLEMENTARY TABLE 3: Developmental changes in mEPSCs and mIPSCs of VIP-INs.

|  | **P3** | **P9** | **P15** | **P30+** |  |
| --- | --- | --- | --- | --- | --- |
| *mEPSCs* | | | | | Statistical test |
| **Amplitude [pA]** | 10.1 ± 1.6  N = 11 | 14.1 ± 2.5  N = 11 | 14.6 ± 3.1  N = 18 | 13.3 ± 3.2  N = 6 | One-way ANOVA with post hoc Tukey’s test  F (3, 42) = 7.017 |
| **P3 vs. P9**  p = 0.0011  **Adjusted**  **p = 0.0056 †** | **P3 vs. P15**  p < 0.0001  **Adjusted**  **p = 0.0004 ‡** | **P3 vs. P30+**  p = 0.0200  Adjusted  p = 0.0891 | **P9 vs. P15**  p = 0.6298  Adjusted  p = 0.9619 | **P9 vs. P30+**  p = 0.5941  Adjusted  p = 0.9495 | **P15 vs. P30+**  p = 0.3365  Adjusted  p = 0.7658 |
| **10-90 Rise Time [ms]** | 1.6 ± 0.6  N = 12 | 1.4 ± 0.47  N = 12 | 1.2 ± 0.3  N = 18 | 1.5 ± 0.2  N = 6 | Kruskal-Wallis with post hoc Dunn’s test |
| **P3 vs. P9**  p = 0.7100  Adjusted  p > 0.9999 | **P3 vs. P15**  p = 0.0345  Adjusted  p = 0.2072 | **P3 vs. P30+**  p = 0.7751  Adjusted  p > 0.9999 | **P9 vs. P15**  p = 0.0879  Adjusted  p = 0.5275 | **P9 vs. P30+**  p = 0.5556  Adjusted  p > 0.9999 | **P15 vs. P30+**  p = 0.0484  Adjusted  p = 0.2901 |
| **Decay Time [ms]** | 5.0 ± 1.8  N = 12 | 5.5 ± 1.1  N = 12 | 5.5 ± 0.9  N = 18 | 6.1 ± 0.5  N = 6 | Kruskal-Wallis with post hoc Dunn’s test |
| **P3 vs. P9**  p = 0.2234  Adjusted  p > 0.9999 | **P3 vs. P15**  p = 0.2425  Adjusted  p > 0.9999 | **P3 vs. P30+**  p = 0.0126  Adjusted  p = 0.0757 | **P9 vs. P15**  p = 0.8689  Adjusted  p > 0.9999 | **P9 vs. P30+**  p = 0.1336    Adjusted  p = 0.8014 | **P15 vs. P30+**  p = 0.0851  Adjusted  p = 0.5107 |
| **Frequency [Hz]** | 0.08 ± 0.06  N = 12 | 0.3 ± 0.1  N = 11 | 0.6 ± 0.4  N = 16 | 0.8 ± 0.3  N = 6 | Kruskal-Wallis with post hoc Dunn’s test |
| **P3 vs. P9**  p = 0.0054  **Adjusted**  **p = 0.0323*** | **P3 vs. P15**  p < 0.0001  **Adjusted**  **p < 0.0001 ‡** | **P3 vs. P30+**  p < 0.0001  **Adjusted**  **p < 0.0001 ‡** | **P9 vs. P15**  p = 0.0800  Adjusted  p = 0.4802 | **P9 vs. P30+**  p = 0.0356  Adjusted  p = 0.2136 | **P15 vs. P30+**  p = 0.4262  Adjusted  p > 0.9999 |
| **Charge Transfer [fC]** | 36.5 ± 16.0  N = 12 | 53.5 ± 17.1  N = 12 | 44.0 ± 9.3  N = 18 | 48.4 ± 9.7  N = 6 | One-way ANOVA with post hoc Tukey’s test  F (2, 33) = 2.088 |
|  |  | **P9 vs. P15**  p = 0.0493  Adjusted  p = 0.1183 | **P9 vs. P30+**  p = 0.4195  Adjusted  p = 0.6950 | **P15 vs. P30+**  p = 0.4607  Adjusted  p = 0.7379 |  |
| *mIPSCs* | | | | | Statistical test |
| **Amplitude [pA]** | 13.6 ± 6.1  N = 10 | 16.3 ± 3,599  N = 11 | 21.1 ± 3.8  N = 17 | 27.0 ± 11.4  N = 6 | Kruskal-Wallis with post hoc Dunn’s test |
| **P3 vs. P9**  p = 0.3176  Adjusted  p > 0.9999 | **P3 vs. P15**  p = 0.0005  **Adjusted**  **p = 0.0033 †** | **P3 vs. P30+**  p = 0.0011  **Adjusted**  **p = 0.0064 †** | **P9 vs. P15**  p = 0.0150  Adjusted  p = 0.0900 | **P9 vs. P30+**  p = 0.0136  Adjusted  p = 0.0815 | **P15 vs. P30+**  p = 0.5120  Adjusted  p > 0.9999 |
| **10-90 Rise Time [ms]** | 2.6 ± 1.0  N = 10 | 2.6 ± 0.8  N = 11 | 1.3 ± 0.3  N = 16 | 1.2 ± 0.6  N = 6 | Kruskal-Wallis with post hoc Dunn’s test |
| **P3 vs. P9**  p = 0.8294  Adjusted  p > 0.9999 | **P3 vs. P15**  p = 0.0005  **Adjusted**  **p = 0.0028 †** | **P3 vs. P30+**  p = 0.0026  **Adjusted**  **p = 0.0158*** | **P9 vs. P15**  p = 0.0001  **Adjusted**  **p = 0.0007 ‡** | **P9 vs. P30+**  p = 0.0012  **Adjusted**  **p = 0.0070 †** | **P15 vs. P30+**  p = 0.7630  Adjusted  p > 0.9999 |
| **Decay Time [ms]** | 33.5 ± 11.9  N = 10 | 23.1 ± 4.4  N = 11 | 17.7 ± 3.9  N = 17 | 13.0 ± 2.1  N = 6 | Kruskal-Wallis with post hoc Dunn’s test |
| **P3 vs. P9**  p = 0.1453  Adjusted  p = 0.8720 | **P3 vs. P15**  p = 0.0002  **Adjusted**  **p = 0.0014 †** | **P3 vs. P30+**  p < 0.0001  **Adjusted**  **p < 0.0001 ‡** | **P9 vs. P15**  p = 0.0314  Adjusted  p = 0.1882 | **P9 vs. P30+**  p = 0.0009  **Adjusted**  **p = 0.0054 †** | **P15 vs. P30+**  p = 0.0727  Adjusted  p = 0.4363 |
| **Frequency [Hz]** | 0.1 ± 0.1  N = 9 | 0.5 ± 0.3  N = 11 | 1.9 ± 1.1  N = 17 | 3.3 ± 2.7  N = 6 | Kruskal-Wallis with post hoc Dunn’s test |
| **P3 vs. P9**  p = 0.0963  Adjusted  p = 0.5779 | **P3 vs. P15**  p < 0.0001  **Adjusted**  **p < 0.0001 ‡** | **P3 vs. P30+**  p < 0.0001  **Adjusted**  **p < 0.0001 ‡** | **P9 vs. P15**  p = 0.0021  **Adjusted**  **p = 0.0124 *** | **P9 vs. P30+**  p = 0.0018  **Adjusted**  **p = 0.0108 *** | **P15 vs. P30+**  p = 0.4081  Adjusted  p > 0.9999 |
| **Charge Transfer [fC]** | 253.4 ± 168.0  N = 10 | 202.9 ± 47.3  N = 11 | 166.8 ± 31.7  N = 17 | 146.5 ± 42.6  N = 6 | One-way ANOVA with post hoc Tukey’s test  F (2, 31) = 4.751 |
|  |  | **P9 vs. P15**  p = 0.0235  Adjusted  p = 0.0593 | **P9 vs. P30+**  p = 0.0080  **Adjusted**  **p = 0.0211*** | **P15 vs. P30+**  p = 0.2842  Adjusted  p = 0.5274 |  |
| *E/I Ratio of synaptic inputs* | | | | | Statistical test |
| **E/I Ratio Synaptic Input Frequency** | 1.3 ± 1.2  N = 10 | 0.9 ± 0.6  N = 10 | 0.3 ± 0.2  N = 14 | 0.3 ± 0.2  N = 6 | Kruskal-Wallis with post hoc Dunn’s test |
|  |  | **P9 vs. P15**  p = 0.0030  **Adjusted**  **p = 0.0090†** | **P9 vs. P30+**  p = 0.0238  Adjusted  p = 0.0715 | **P15 vs. P30+**  p = 0.8985  Adjusted  p > 0.9999 |  |
| **E/I Ratio Synaptic Input Charge Transfer** | 0.2 ± 0.1  N = 10 | 0.3 ± 0.1  N = 11 | 0.3 ± 0.1  N = 17 | 0.3 ± 0.1  N = 6 | One-way ANOVA with post hoc Tukey’s test  F (2, 31) = 1.223 |
|  |  | **P9 vs. P15**  p = 0.9102  Adjusted  p = 0.9929 | **P9 vs. P30+**  p = 0.1932  Adjusted  p = 0.3897 | **P15 vs. P30+**  p = 0.1401  Adjusted  p = 0.2982 |  |

SUPPLEMENTARY TABLE 4: Detailed statistical information of developmental changes in eEPSCs and eIPSCs of VIP-INs.

|  | **P9** | **P15** | **P30+** |  |
| --- | --- | --- | --- | --- |
| *eEPSCs* | | | | Statistical test |
| **Amplitude [pA]** | 23.8 ± 11.9  N = 6 | 20.8 ± 6.7  N = 9 | 57.3 ± 22.0  N = 10 | Kruskal-Wallis with post hoc Dunn’s test |
|  | **P9 vs. P15**  p = 0.9088  Adjusted p >0.9999 | **P9 vs. P30+**  p = 0.0031  **Adjusted**  **p = 0.0093 †** | **P15 vs. P30+**  p = 0.0006  **Adjusted**  **p = 0.0017 †** |  |
| **Decay Time [ms]** | 5.1 ± 0.9  N = 6 | 5.6 ± 1.0  N = 9 | 5.3 ± 1.4  N = 10 | One-way ANOVA with post hoc Tukey’s test  F (2, 22) = 0.3640 |
|  | **P9 vs. P15**  p = 0.4168  Adjusted p = 0.6903 | **P9 vs. P30+**  p = 0.7351  Adjusted p = 0.9375 | **P15 vs. P30+**  p = 0.5783  Adjusted p = 0.8403 |  |
| **PPR** | 0.7 ± 0.3  N = 6 | 1.2 ± 0.3  N = 9 | 0.9 ± 0.2  N = 10 | One-way ANOVA with post hoc Tukey’s test  F (2, 22) = 8.563 |
|  | **P9 vs. P15**  p = 0.0009  **Adjusted**  **p = 0.0026 †** | **P9 vs. P30+**  p = 0.2891  Adjusted p = 0.5323 | **P15 vs. P30+**  p = 0.0045  **Adjusted**  **p = 0.0120 *** |  |
| *eIPSCs* | | | | Statistical test |
| **Amplitude [pA]** | 138.3 ± 75.1  N = 10 | 238.1 ± 124.9  N = 8 | 383.7 ± 155.8  N = 10 | One-way ANOVA with post hoc Tukey’s test  F (2, 25) = 10.05 |
|  | **P9 vs. P15**  p = 0.0994  Adjusted p = 0.2208 | **P9 vs. P30+**  p = 0.0002  **Adjusted**  **p = 0.0004 ‡** | **P15 vs. P30+**  p = 0.0196  **Adjusted**  **p = 0.0496 *** |  |
| **Decay Time [ms]** | 21.0 ± 6.2  N = 10 | 16.1 ± 6.7  N = 8 | 11.2 ± 4.9  N = 13 | One-way ANOVA with post hoc Tukey’s test  F (2, 28) = 8.049 |
|  | **P9 vs. P15**  p = 0.0912  Adjusted p = 0.2052 | **P9 vs. P30+**  p = 0.0004  **Adjusted**  **p = 0.0012 †** | **P15 vs. P30+**  p = 0.0679  Adjusted p = 0.1576 |  |
| **PPR** | 0.9 ± 0.2  N = 10 | 1.0 ± 0.3  N = 8 | 0.6 ± 0.2  N = 13 | One-way ANOVA with post hoc Tukey’s test  F (2, 28) = 8.988 |
|  | **P9 vs. P15**  p = 0.6013  Adjusted p = 0.8581 | **P9 vs. P30+**  p = 0.0022  **Adjusted**  **p = 0.0060 †** | **P15 vs. P30+**  p = 0.0009  **Adjusted**  **p = 0.0025 †** |  |
